# Enlarging viral mutation estimation: a view from the distribution of mutation rates

**DOI:** 10.1099/mic.0.001717

**Published:** 2026-08-12

**Authors:** Taima Naomi Furuyama, Isabel Maria Vicente Guedes de Carvalho, Luiz Mário Ramos Janini, Fernando Antoneli

**Affiliations:** 1 Laboratório de Virologia, Instituto Butantan, São Paulo, SP, Brazil; 2 Departamento de Microbiologia, Imunologia e Parasitologia, Departamento de Medicina, Laboratório de Retrovirologia, Universidade Federal de São Paulo, São Paulo, São Paulo, Brazil; 3 Centro de Bioinformática Médica, Universidade Federal de São Paulo, São Paulo, São Paulo, Brazil

**Keywords:** Bayesian inference, information dimension, information theory, mutation rate distribution, mutational rates, RNA viral populations, single-round replication cycle

## Abstract

The problem of empirical estimation of mutation rates is fundamental to the understanding of viral evolution. The estimation of viral mutation rates is based on varied and often complex methods carried out through experiments essentially designed to count mutation frequencies. Mutation rates are defined as the probabilities of nucleotide substitutions, typically reported as a single number in units of mutation (substitution) per base (nucleotide) per replication cycle or per cell infection, depending on the replication mode of the virus. Even more, the uncertainty quantification of these estimates is so difficult that it is rare to find it reported in the literature. The values for the same virus reported in literature fall within a broad range, sometimes spanning two orders of magnitude. For instance, the mutation rates range from 10^−8^ to 10^−6^ mutations per base per cell infection for DNA viruses and from 10^−6^ to 10^−4^ mutations per base per cell infection for RNA viruses. The main issue here is that for the same viruses, there are several mutation rate estimates with different measuring units. In this paper, we propose an alternative perspective on the estimation of mutational rates, which avoids the use of serial passages. Our approach leverages the large amount of sequencing data produced by high-throughput sequencing technologies coupled to an experimental design that performs a single replication cycle from an initial clonal viral population. We propose to replace the single numeric mutation rate with a distribution of mutation rates (DMRs), together with a procedure to implement the estimation of this distribution from sequencing data, and show that it can be estimated from sequencing data. Even though the focus of this paper is the development of the approach centred on the DMR, it is straightforward to produce point and interval estimates of the mutation rates, including uncertainty quantification. In addition to the estimation of the DMR, we provide a theoretical characterization of it, as being well approximated by a log-normal distribution. Finally, we illustrate how the DMR can be used in non-trivial ways to study global properties of the genome. Namely, using methods of information theory, we show that there is a remarkable ‘scaling invariance’ related to the procedure of localization of the DMR from the whole genome to its genomic subunits.

## Introduction

A key concept in viral population knowledge is mutation rates. It is necessary to understand the processes where a drug can act and its risks/side effects, like the pro-drug molnupiravir that leads SARS-CoV-2 to lethal mutagenesis through nucleoside analogues or hepatitis C virus (HCV) NS5A and NS5B inhibitors [1–3]. Moreover, influenza virus strain recommendations for seasonal vaccination worldwide and COVID-19 variant classification to identify the impact risk on the epidemiological situation are public health decision tools based on the dynamics of viral populations, including their mutations [4, 5].

Spontaneous mutations are mutations that occur in the absence of exogenous influence. They may be due to errors made by DNA polymerases during replication or repair, errors made during recombination, the movement of genetic elements or spontaneously occurring DNA damage. The rate at which spontaneous mutations occur is a fundamental evolutionary parameter.

It is important to distinguish between the rate at which spontaneous mutations occur and the rate at which genetic changes accumulate in a surviving lineage. The ‘spontaneous mutation rate’ reflects the probability of a change in genome sequence between a parent and its offspring. It is the compound result of several molecular processes that introduce errors during the transmission of genetic information. However, only those mutations in lineages that persist – typically in the face of selection – contribute to the ‘substitution rate’. Consequently, the frequency of a mutant allele in a population generally does not equal the rate at which the corresponding mutational event occurs.

The mutation rate of a virus is the expected number of mutations that a viral particle will sustain during its replication cycle. In empirical experiments designed to estimate spontaneous mutation rates, it is very common to measure mutation frequencies which, together with other information, are used to compute a single number *μ*, the *mutation rate estimate* [6, 7]. Broadly speaking, there are two classes of methods for determining *μ* [8]: by mutation frequency, that is, as the mean, median or proportion of some sampling-counting scheme [9, 10], or by a fluctuation test, that is a method for mutation rate estimation derived from the classic Luria–Delbrück experiment [11–13].

Accurate and precise estimation of mutation rates has proven challenging due to the difficulty in sampling genomic data and counting mutation frequencies, as well as the numerous possibilities for combining mutation frequencies and population history to calculate mutation rates. Tests for accumulation of mutations usually involve culturing viral populations for generations of replicative cycles, serial passages with transmission bottlenecks and/or plate-to-plate transfers. These practices already impose a selection process on the viral population, i.e. *de novo*, lethal and deleterious mutations may not be detected in the majority viable population [8]. Fluctuation tests, such as those performed in [13], are free from reverse transcription or PCR errors and may exhibit less bias against lethal and deleterious mutations. Furthermore, the events detected may be context-dependent, and the analysed mutational spectrum may be limited [8].

Because of these difficulties, the literature is fraught with apparently disparate estimates – typically spanning one or two orders of magnitude – not to mention the use of distinct units: m/b/r (mutations/base/replication cycle) and m/b/c (mutations/base/cell infection). See [10] for a discussion on the relation of these two units and how to convert one to the other. For instance, it is possible to find in the literature the following estimates for three important viruses: (i) HIV, 5.4×10^−5^ m/b/r and 3.0×10^−4^ m/b/r (purified HIV-1 RT) [14], 3.4×10^−5^ m/b/r [15], 2.4×10^−5^ m/b/c [10], 1.4×10^−5^ m/b/r and 5.9×10^−4^ m/b/r (purified HIV-1 RT) [16], 6.2×10^−5^ m/b/c [17]; (ii) HCV, 1.2×10^−4^, 2.5×10^−5^, 2.0×10^−5^ and 3.5×10^−5^ m/b/c; (iii) influenza virus A, 7.1×10^−6^, 4.5×10^−5^, 3.9×10^−5^ and 3.1×10^−5^ m/b/c [18]. Mutational rates are affected by many factors according to the virus. It is relevant to consider the enzyme fidelity, whether the polymerase or the exonuclease, the replication process and its interaction with the immune response system of the host, nucleic acid spontaneous damage, access to post-replicative repair, number of available nucleotides, quality and structure of emerging templates [18].

In this paper, we propose an alternative perspective on the estimation of spontaneous mutation rates. Our approach leverages the large amount of sequencing data produced by high-throughput sequencing technologies coupled with an experimental design that performs a single replication cycle from an initial clonal viral population [19]. Although we are not the first to propose the use of high-throughput sequencing for the estimation of viral mutation rates [20–23], we depart from them regarding the estimation method. Moreover, we expect to bypass the difficulties with sampling/counting mutation frequencies and account for all mutation occurrences, including possible *de novo*, neutral, deleterious and lethal mutations [8].

Therefore, to account for the wide range of mutation rates that can occur, we treat all of them on an equal footing as a ‘distribution of mutation rates (DMR)’ rather than as a single numerical value. As we will see, the DMR, as defined here, simultaneously comprehends two ‘dimensions’ of the genetic variability, across the population and along the genome. We should mention that other authors already considered the notion of DMRs in different contexts: [24] for viral populations and [25] for microbial populations to account for the wide range of mutation rates that can occur. Herein, we extend the methods of [26] to include a procedure to estimate the DMR associated with the sequencing data mentioned above and show that it has a specific statistical characterization.

It is fundamental to keep in mind that our method strongly relies on the occurrence of a single replication cycle and, therefore, we can only make inferences about spontaneous mutation rates. In other words, we cannot conclude anything about substitution rates, and this distinction is fundamental for the interpretation of our results. Another important caveat to be concerned with is that in this paper, we will restrict ourselves to discuss only RNA viruses. First, because this is the only case that we have studied so far. Second, due to some particularities of the method, we only can ensure that it can be reliably applied to other RNA viruses. One critical assumption behind our rationale is that the organism is expected to have reasonably high mutation rates – on the order 10^−7^ or higher. This excludes even certain DNA viruses [6, 7, 27–29].

Mutational rates defined as a single numerical value can hide potentially useful information. A viral population is dynamic and uses genomic strategies to reach its balance between mutations, drift and selection according to a fitness landscape. This ‘population’ viewpoint allows for a more comprehensive framework for the representation of the mutational dynamics. In fact, the notion of DMR fits very well within several modelling approaches to describe the evolutionary dynamics of viral populations [30–37].

## Methods

All the computations were carried out using the statistical software R with the RStudio IDE [38, 39]. The R package ModeEst (Mode Estimation, version 2.4.0 [40]) was used to perform the mode estimation. The R package poweRlaw (Analysis of Heavy Tailed Distributions, version 0.80 [41, 42]) was used to perform the log-normal fit and the hypothesis test if the data are generated or not from a log-normal distribution using the PLFit algorithm of Clauset *et al*. [43]. The R package Mass (Modern Applied Statistics with S, version 7.3–54 [44]) was used to compute the Q-Q plots and the curve fitting for the univariate continuous distributions.

### Theoretical background: the DMRs

The motivation to consider the DMRs is to reflect the fluctuations of mutation rates across co-existing individuals, for each site in the genome. Therefore, one is led to consider the *mutation rate per site* as a random variable. Because of this shift in perspective, our approach allows one to see the effects of population heterogeneity, that is, the variation in the mutation probabilities per site. This approach contrasts with many previous studies that compared the presence versus absence of a ‘mutator’ type, thus changing the mean and the variance of the mutation rate [24].

In [25], the authors propose that the DMR associated with *bacteria* is a log-normal probability distribution in order to satisfy a set of attributes. Here, we adapt the proposal of [25] to the context of RNA *viruses* as follows:

1. most mutation rates are low and very close to 0, around 10^−4^ m/b/r,
2. none of them are zero, and almost none are extremely low, e.g. ≤10^−6^ m/b/r,
3. very few of them are higher.

This suggests that the DMR associated with an RNA virus could be a log-normal probability distribution. However, we will not assume that the DMR is modelled by a log-normal probability distribution, as [24, 25]. Instead, we will empirically define the DMR and show that it is well-approximated by a log-normal probability distribution.

Since a log-normal distribution has a long tail of few high-rate mutations, it is expected that a DMR has mean>median>mode. This means that using the DMR to obtain point estimates of the mutation rate as median or mean values can give higher values than traditionally obtained, as the usual methods for estimating mutation rates are sensitive to extreme cases encountered in preferred mutation sites.

There is a connection between the DMR and the *Luria-Delbrück distributions* that usually appear in fluctuation tests. First, we start by observing that the DMR is connected to the per-*loci* distribution of mutations occurring on a genome in a single generation (see [24]). Assume that the mutation rate is uniform along *loci* within one individual, and that given this rate, mutations occur independently among *loci*. Then, the number of new mutations *j* that arise ‘simultaneously’ (i.e. in one generation) on an individual with *n loci* and with realized mutation rate *μ* follows a *binomial distribution*. In the limit as *n* → ∞ and *μ* → 0, such that *nμ*=λ, one obtains the *infinite-locus model* in which every new mutation occurs at a unique site. Then, the number of mutations occurring in an individual with per-genome mutation rate *λ* follows a *Poisson distribution*. Finally, by averaging against the DMR, one obtains a *mixed Poisson distribution* [45], which is the corresponding *Luria-Delbrück distribution*.

### Estimation of the DMRs

The results of this paper heavily rely on the two previous papers of our group, namely, [19, 26]. The experimental protocol and the sequencing protocol are detailed in [19]. The bioinformatics analysis and the statistical methods are explained in [26]. The three papers ([19, 26] and the present one) complement each other. In the following, we will briefly recall the relevant information from [19, 26] necessary for the implementation of the new methods introduced here. In order to carry out all the analysis for the DMR estimation, one only needs the publicly available data published in [19], regarding the mutation rates per site from a set of single replication cycle experiments with HIV-1.

**Fig. 1.**
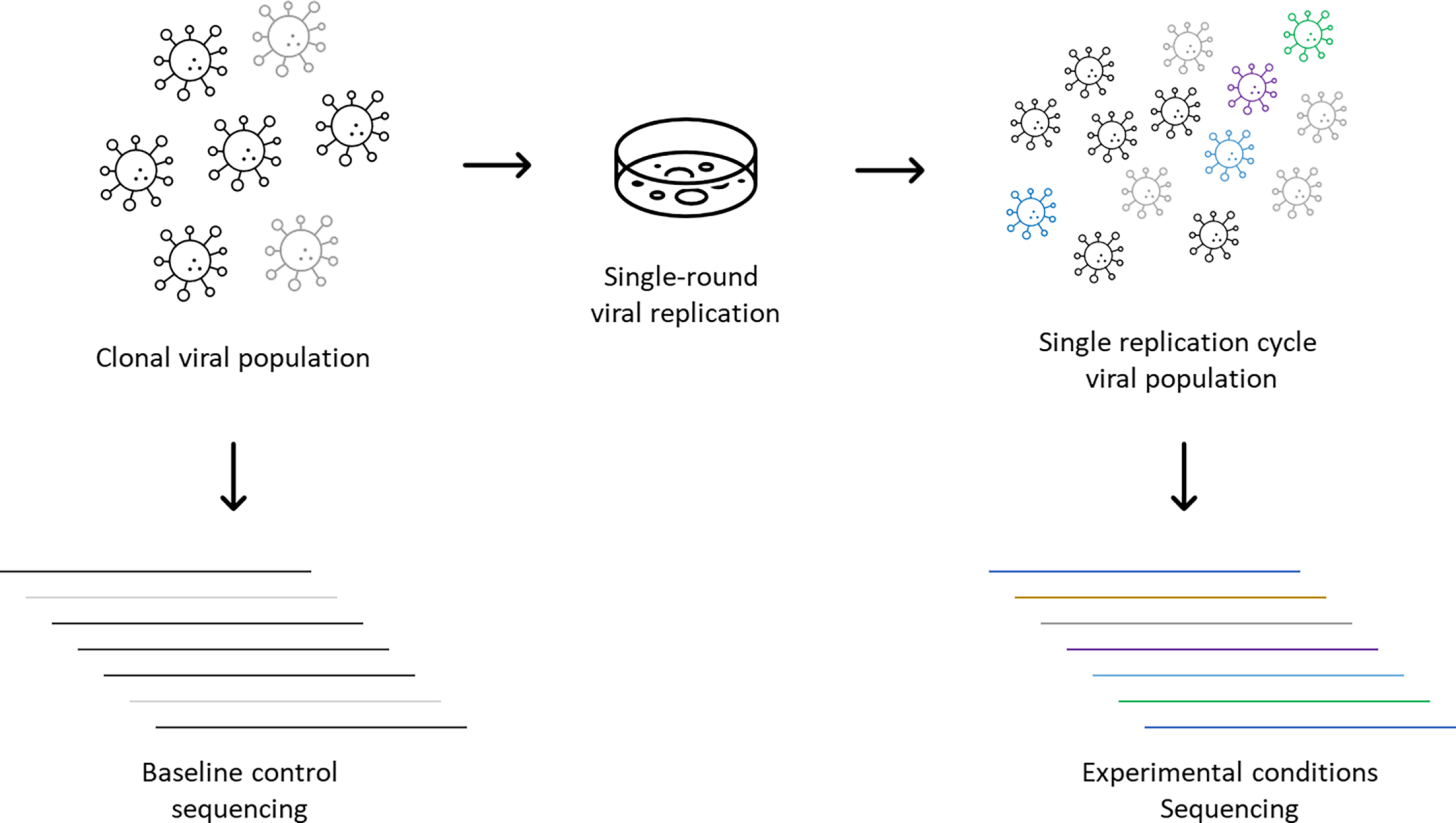
The diagram shows a summary of the sequencing data. The initial viral population, formed from a defective viral type for the *env* gene, was replicated in a single round. As a clonal population, the diversity shown in the sequences is low (represented by the colours). From there, the viral progeny composed a population with its own mutation occurrence, showing an increased diversity. The progeny population was sequenced, and the analysed data were provided.

In a very summarized way, several cell culture procedures were performed through transfection by two cellular states (stimulated/S and non-stimulated/NS), two types of cells (TCD4 cells - CD4+ T lymphocytes - or PBMCs - Peripheral Blood Mononuclear Cells) and two types of pseudoviruses (CCR5/R5 or CXCR4/X4). The viral sequences were obtained from the proviral genome using the SOLiD^™^ technology. The assembly was obtained by mapping the reads against a reference, and the whole genome (without the LTR regions - Long Terminal Repeat regions) is given by positions 791 to 9,085. A total of seven experimental conditions and a baseline control (BC) experiment were considered: (1) NS_CD4_R5, (2) NS_CD4_X4, (3) NS_PBMC_R5, (4) NS_PBMC_X4, (5) S_CD4_R5, (6) S_PBMC_R5, (7) S_PBMC_X4 and (8) BC. A putative experimental condition ‘S_CD4_X4’ is missing because the sequencing was not successful in this case. The BC is obtained by sequencing the original plasmidial clone DNA used to construct the pseudoviruses. That is, the BC represents the viral DNA *before* the infection (see Fig. 1 and [19] for details).

Regarding the data analysis, we adopted an *empirical Bayes* approach [46] (see also Appendix A in the Supplementary Material for an updated version of the procedure described in [26]). There are two main advantages of using the empirical Bayes approach for the problem of DMR estimation. First, the inference provides a conditional posterior probability distribution over the multinomial parameters, and thus one can interpret all point estimates as probabilities. Second, the data are naturally split into a ‘prior condition’ (before replication) and a ‘posterior condition’ (after one replication cycle), fitting properly into a Bayesian framework. See [26] for a detailed discussion on the advantages and drawbacks of the main approaches to the problem of estimation of multinomial parameters. In the approach proposed by [26], the pre-infection BC (the sequencing data from the plasmidial clone sample) is used to compute the *prior distribution*. Using *Bayes formula*, the data of the post-infection experimental conditions are combined with the *prior distribution* to compute the *posterior distribution*. Finally, the BC is used to account for the sequencing error rates, as reported by the sequencer manufacturer, as well (see [26] and Appendix A in the Supplementary Material for details).

The analysis generates estimates for the *multinomial probability distribution* per site, that is, a vector of real numbers (p_A, p_T, p_G, p_C) between 0 and 1, with p_A+p_T+p_G+p_C=1 for each site in the genome. Hence, for each experimental condition and the BC, there is a list of multinomial probability distributions per site. These distributions represent the variability of the mutation rate per site across the population.

From the multinomial probability distributions, the complementary probability at each site is given by


$$p_{comp}=1-\max\left\{p_{A},\;p_{T},\;p_{G},\;p_{C}\right\}$$

**Fig. 2.**
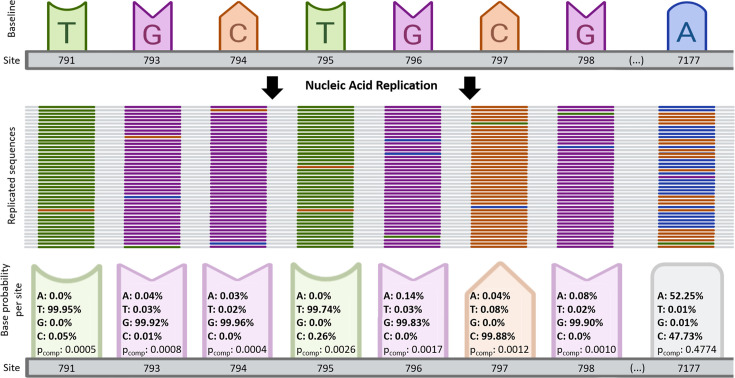
Diagram showing the empirical Bayes approach to the inference of bases from the experimental data. Data from https://doi.org/10.1371/journal.pone.0139037.s001/nonstimulated-CD4-X4.xls.

The interpretation of $p_{comp}$ is that it is the sum of the probabilities of the three remaining nucleotides after the most probable is removed. The complementary probability $p_{comp}$ is an overall indicator of the fluctuation of the possible nucleotide at each site. A higher $p_{comp}$ indicates that two or more nucleotides are highly likely at that position. A lower $p_{comp}$ indicates that a single nucleotide is overwhelmingly prevalent at that position. See Fig. 2.

The DMR is defined as the empirical distribution generated by the set of the complementary probabilities $\left\{p_{comp}\right\}$ along the genome – after filtering out sites that satisfy exclusion criteria. The exclusion criteria are used to discard sites that (i) are extremely variable and (ii) such that the uncertainty of the estimate of max{p_A, p_T, p_G, p_C} is below the accuracy of the method (see [26]). Sites that satisfy (ii) are such that $\left\{p_{comp}\right\}$ is so small that we cannot distinguish it from 0. The BC is used again at this point. Item (i) is obtained by excluding sites whose complementary probability was above 6.0×10^−3^ (97.5% quantile of the raw data of the BC), and item (ii) is obtained by excluding sites whose complementary probability was below 10^−4.2^≈6.3×10^−5^ (0.25% quantile of the raw data of the BC).

Since we expect that the empirical DMR is well approximated by a log-normal distribution, it is natural to transform the original data $\left\{p_{comp}\right\}$ to a logarithmic scale, which should follow a normal or Gaussian distribution. We use the natural logarithm transformation s=-ln⁡(p) also known as the *s-score* or *surprisal*, or *self-information* [47–51]. Even though the log-*transformed* DMR, or log-DMR for short, looks more familiar (since it will be approximately Gaussian), the interpretation and point estimates only make sense on the original scale of the data.

Therefore, the *empirical* DMR and the corresponding log-DMR estimated here are ‘trimmed’ empirical distributions. The quantile values in the exclusion criteria were chosen to ensure maximum retention of informative data while also providing a robust version of the DMR. In fact, about 95% of the sites from the raw data were retained in all conditions, resulting in ~7,800 sites per genome. Recall that our genomic coordinates range from 791 to 9,085 (HIV-1 whole genome without LTR regions), and so we have a total of 8,294 sites. That is, about 400–500 sites were excluded from each condition.

The empirical DMR and the corresponding log-DMR are univariate empirical distributions and hence can be visualized in a simple 2D plot. Moreover, several measures of central tendency and dispersion, such as mean, median, mode, range, upper and lower quartiles, variance and standard deviation, can be computed for these distributions. In particular, the mean, median and mode can serve as point estimates for the mutation rate seen as a single numerical value, for the sake of comparison with the estimates from the literature.

We do not compute the empirical distribution associated with the BC data, simply because this empirical distribution is not a DMR. In fact, since the BC data are obtained by sequencing the plasmidial clone, instead of reflecting the action of the virus replication mechanism, it reflects the copying machinery of the bacteria used to propagate it.

### Characterization and properties of the DMRs

We performed a pipeline of exploratory data analysis on the empirical DMRs obtained from the seven experimental conditions and the BC. The pipeline consists of two main steps: (a) pairwise two-sample Kolmogorov–Smirnov tests comparing the DMRs from the seven experimental conditions and the BC to assess the similarity or dissimilarity between them, and (b) an analysis of the impact of the exclusion of invariant sites.

#### Pairwise two-sample Kolmogorov–Smirnov test

We performed a pairwise two-sample Kolmogorov–Smirnov test with all experimental conditions and the BC. The goal was to verify whether the empirical DMRs were the same or not. Since our data come from multiple experiments comprising distinct experimental conditions, we want to verify if these conditions affect the DMR. The test was performed for all possible pairs of 7 conditions, resulting in a total of K=(7×6)/2=21 tests. We assumed a significance level of *α*=0.05 per test; hence, using the *Bonferroni adjustment*, we had a total significance level of *α*_B_=0.002. Here, we need to apply the Bonferroni adjustment because we are performing several pairwise tests on the same dataset, and we want to avoid Type I error. This is similar to the post-hoc pairwise comparison performed after a significant one-way ANOVA is obtained, to find out which specific groups differ. The post-hoc test systematically compares each pair of groups to find where the significant differences lie. It includes an adjustment to the *p*-value to BC the increased risk of a Type I error (a false positive) from conducting many tests at once.

#### Impact of the exclusion of sites

Recall that we exclude sites that (i) are extremely variable and (ii) had uncertainty below the accuracy of the estimation method. It is worth mentioning that the hyper-mutated sites, excluded by criterion (i), were already analysed in [19]. The sites that satisfy the exclusion criterion (ii) can be considered as the ‘invariant sites’, since for them, $p_{comp}$ is statistically indistinguishable from 0.

Therefore, we can investigate whether there are patterns of invariant sites that are recurrent among the experimental conditions. First, we check if the invariant sites are concentrated in some region or randomly spread along the genome. Then, we compute the sets of invariant sites of each experimental condition and determine the invariant sites that are excluded from more than one experimental condition. For those invariant sites that appear more than once, we count how many conditions they were present. Finally, using this information, we can search for possible preferred mutation regions.

In order to verify whether a significant number of consecutive sites are excluded when applying the exclusion criteria, we compute the length and position of the gaps left after the exclusion of sites.

#### Scale invariance and information dimension

The idea here is to investigate how the empirical DMR behaves when one considers not only the whole-genome data but also the data from its subunits (e.g. genes and fragments). The motivation for this analysis is to assess what happens to the DMR when we change the genomic region where it is defined. Does it remain close to a log-normal? If this turns out to be true, then it can be seen as a kind of ‘scaling invariance’, where the ‘scale’ here refers to the length of the genomic region considered and the ‘invariance’ refers to the preservation of the DMR ‘shape’. This is a very useful property because one no longer needs to obtain whole-genome sequences to estimate the DMR; a sufficiently long region would be enough.

We first investigate how the empirical DMR behaves under ‘localization’ to biologically significant genomic subunits: the genes and smaller fragments, up to a length of 50 nucleotides. We compare the main statistical summary (i.e. the mode, the median and the mean) change as we decrease the fragment size all the way to 50 nucleotides. Then, we perform a simulation analysis where we pick random fragments of decreasing sizes, starting with fragments of 1,000 nucleotides, down to 50 nucleotides, in steps of 25. We simulate 1,000 fragments for each size and compute the mean of the log-DMR for each of them. With a list of 1,000 mean values for each fragment size, we calculated the *range* (i.e. the difference Δ=max − min) and the *mean μ* (i.e. the mean of the mean values). By plotting these two functions against the fragment size, we can investigate the behaviour of the DMR under localization.

Once confirmed, ‘scale invariance’ defines the pattern – namely, the log-normal shape of the DMR – that repeats at different scales. We want to quantify the ‘density’ and ‘complexity’ of that pattern. For that, we resort to the theory of information, more precisely, the notion of *information dimension* associated with a probability distribution. The idea of *information dimension* is to quantify how the probability distribution associated with a given system behaves when some scaling parameter changes. Formally, it is given by the growth rate of the Shannon entropy, associated with successively finer discretizations, referred to as *coarse-grained approximations* [52].

The intuition behind the definition of information dimension is the following. If we interpret the elements of the coarse-grained approximation at a scale *ε* as the size of a ‘box’, then we can interpret the associated Shannon information *S*(*ε*) as the average information ‘per box’. The number *d*, called *information dimension*, is the exponent of the ‘box size’, when *ε* is small enough, corresponding to the average information needed to identify an occupied box, that is, exp *S*(*ε*) ≈ *ε*^−*d*^. In this sense, the information dimension measures how the distribution associated with the coarse-grained approximation ‘scales’ with respect to the size of the coarse-graining. The exponential of the Shannon entropy exp *S* is called *perplexity* in the fields of machine learning and statistical modelling and is called *diversity index of order* 1 in the fields of ecology and demography [53].

We provide a brief review of the main concepts related to information theory and information dimension that will be necessary for our analysis in the Supplementary Material, Appendix C.

## Results

In this section, we describe the estimation of the DMRs and the performance of several tests and comparisons. In the first subsection, we constructed the empirical DMR for the seven experimental conditions and the ‘aggregate condition’, defined by averaging (geometric mean) the seven experimental conditions over each site. We started by showing that the DMRs are log-normal distributions by fitting the tail of the DMR to a theoretical log-normal distribution and comparing the log-DMR with a theoretical Gaussian distribution. The skewness estimation showed that the small deviation of the log-DMR from Gaussian is within the acceptable margin. We conducted some exploratory data analysis. First, we performed a pairwise two-sample Kolmogorov–Smirnov test comparing the seven DMRs against each other to see if the differences in the setup of the experimental conditions could affect the DMR. Second, we evaluated the impact of the exclusions of invariant sites. The scale invariance was a surprising empirical finding that showed up when we were investigating the possibility of considering DMR associated with genomic subunits, e.g. genes. We investigated, by simulation, the impact of localizing the DMR to fragments of decreasing length. Lastly, the application of information dimension is a natural approach to model a system with scale invariance. By dividing the genome into smaller fragments, we were able to confirm that the scale invariance supports the hypothesis that mutations are uniformly and randomly distributed over the genome.

### Distributions of mutation rates

We estimated the empirical DMRs for all seven experimental conditions. We defined the *aggregate condition* by averaging (geometric mean) the seven experimental conditions over each site and estimated its DMR, as well. In the following, we use the DMR of the aggregate condition to illustrate the method and other complementary analyses. The same analysis was carried out for all seven experimental conditions, and the results are very similar (we will not show all cases here; see the Supplementary Figures).

**Fig. 3.**
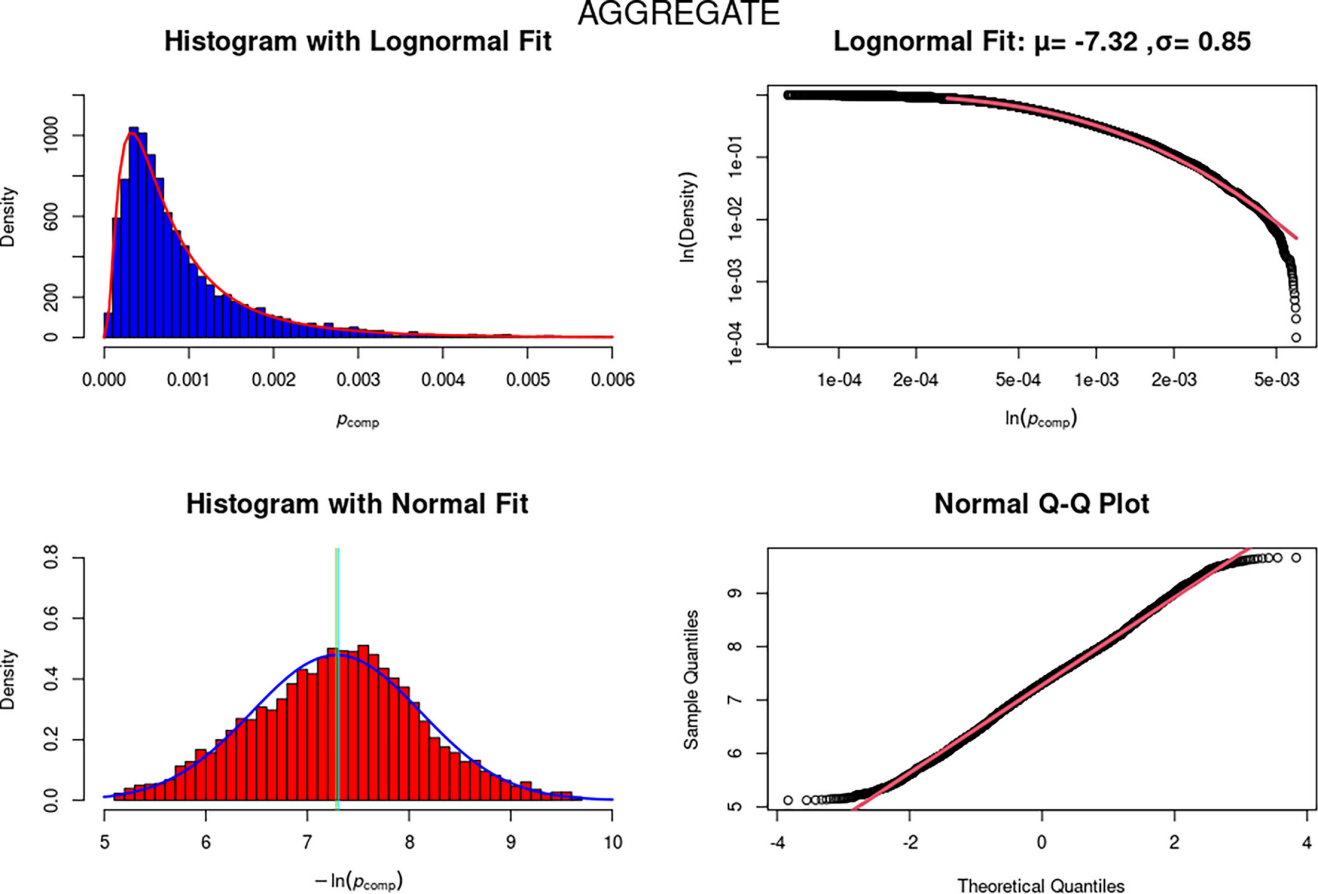
Different views of the empirical DMR (aggregate condition). (Top left) Histogram of the empirical DMR, with the theoretical log-normal distribution (red curve) superimposed. (Bottom left) Histogram of the empirical log-DMR, with the theoretical normal distribution (blue curve) superimposed. Green and cyan vertical lines indicate mean and median, respectively. (Top right) Empirical cumulative DMR in ln-ln scale with log-normal tail fit (red curve). (Bottom right) Normal Q-Q plot of the empirical log-DMR with the expected line if the empirical distribution equals the theoretical normal distribution (red line).

Fig. 3 has four panels showing different aspects of the DMR for the aggregate condition. The top left panel shows the histogram of the empirical DMR. The bottom left panel shows the histogram of the corresponding log-DMR (see Fig. S1 for all conditions). The bottom right panel shows the normal Q-Q plot of the log-DMR (see Fig. S2 for all conditions). The top right panel shows the corresponding cumulative DMR ln-ln scale with a log-normal tail fit [42]. All these plots illustrate that the empirical DMRs indeed are very well approximated by a log-normal distribution. The histograms for the DMR and the log-DMR, on the left, are superimposed by the corresponding hypothesized theoretical distributions (continuous curve). The log-normal tail fit and the normal Q-Q plot show that the empirical DMR is very well approximated by a theoretical log-normal and normal distributions, respectively.

Finally, we performed the bootstrap test of [43] for the (null) hypothesis (H_0_) that the data are generated from a log-normal distribution against the (alternative) hypothesis (H_1_) that the data are not generated from a log-normal distribution, at the 99% significance level [41–43]. In all cases, we obtained *p*>0.9, indicating that the null hypothesis cannot be rejected, and hence, a log-normal distribution is a good approximation to the empirical DMR. It is important to point out that other types of distribution can explain the same data, as well. This possibility is not excluded by the fact that the null hypothesis was not rejected.

Table 1 shows some summary statistics of the DMRs for all seven experimental conditions and the aggregate. As expected from a log-normal distribution, we always have that mode<median<mean. This numerical information can be used to extract point and interval estimates of mutation rate from the DMR. This is important for comparison with results from the literature. The three basic measures of central tendency, mode, median and mean, provide point estimates. The mode and the median are more robust than the mean, which is very sensitive to variation in the tail of the distribution. One advantage of the mean is that one can use the standard error to construct confidence intervals. Here, the standard error can be obtained as the standard deviation divided by the square root of the number of sites used to construct the DMR. Another possibility is to use the median as a robust point estimate and the interval associated with the interquartile range (IQR), defined as [Q_1_ − Q_3_], as a robust interval estimate.

**Table 1.** Summary statistics of the empirical DMR for the seven experimental condition and the aggregate condition. Min.: minimum value; Q^_1_^: first quartile; Q_3_: third quartile; Max.: maximum value; R.S.: proportion of retained sites, i.e., the sites that were considered for the analysis

Condition	Min.	Q_1_	Mode	Median	Mean	Q_3_	Max.	R.S.
NS_CD4_R5	6.38×10^−5^	3.85×10^−4^	3.93×10^−4^	6.61×10^−4^	9.39×10^−4^	1.17×10^−3^	5.97×10^−3^	95%
NS_CD4_X4	6.33×10^−5^	3.74×10^−4^	3.99×10^−4^	6.51×10^−4^	9.40×10^−4^	1.18×10^−3^	5.87×10^−3^	93%
NS_PBMC_R5	6.34×10^−5^	3.94×10^−4^	4.37×10^−4^	6.64×10^−4^	9.50×10^−4^	1.17×10^−3^	5.99×10^−3^	95%
NS_PBMC_X4	6.35×10^−5^	3.83×10^−4^	3.95×10^−4^	6.45×10^−4^	9.17×10^−4^	1.13×10^−3^	5.99×10^−3^	95%
S_CD4_R5	6.32×10^−5^	3.49×10^−4^	3.22×10^−4^	6.13×10^−4^	8.80×10^−4^	1.10×10^−3^	5.98×10^−3^	93%
S_PBMC_R5	6.31×10^−5^	3.52×10^−4^	3.98×10^−4^	5.98×10^−4^	8.89×10^−4^	1.09×10^−3^	5.95×10^−3^	94%
S_PBMC_X4	6.33×10^−5^	3.53×10^−4^	4.03×10^−4^	6.20×10^−4^	9.36×10^−4^	1.14×10^−3^	5.97×10^−3^	94%
AGGREGATE	6.34×10^−5^	3.70×10^−4^	3.92×10^−4^	6.36×10^−4^	9.22×10^−4^	1.14×10^−3^	5.96×10^−3^	94%

**Fig. 4.**
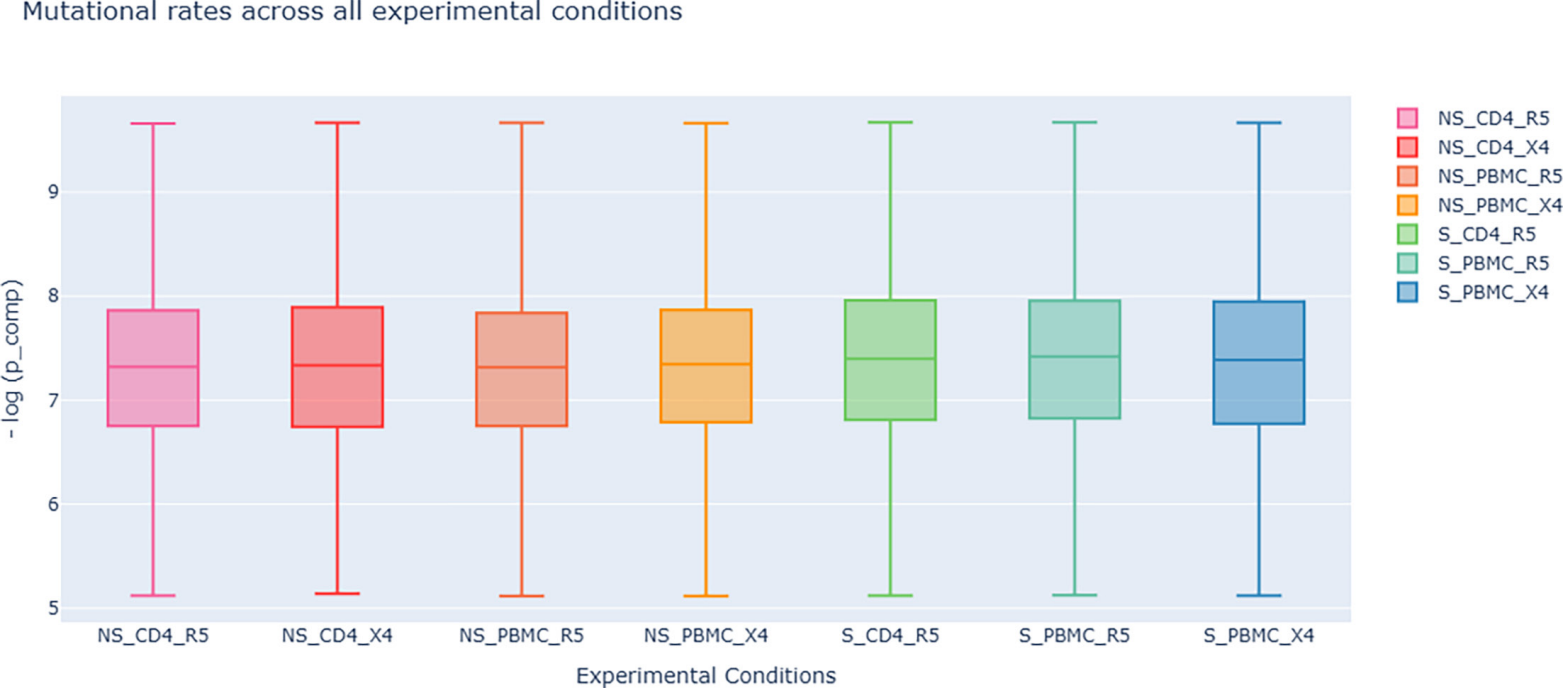
Box plots of the log-DMRs for the seven experimental conditions. The horizontal side of the box indicates the first quartile, the median and the third quartile, respectively. The whiskers were set to be the minimum and the maximum.

Passing to the log-DMRs, Fig. 4 shows the box plots of the log-DMRs for the seven experimental conditions. Since these distributions are approximately Gaussian, we expect that the boxes are divided into almost equal parts and the whiskers have almost equal length.

We wanted to quantify how much divergence there is between the mode, the median and the mean, because this divergence indirectly quantifies how much the log-DMR deviates from a theoretical Gaussian distribution in the bulk (i.e. away from the tails).

The skewness of a distribution is a measure of the asymmetry of the distribution about its mean. If the distribution is both symmetric and unimodal, then mode=median=mean. More generally, for unimodal distributions, the skewness value can be positive, zero, negative or undefined. Roughly speaking, negative skewness indicates that the left tail is longer and the mass of the distribution is concentrated on the right side; positive skewness indicates that the right tail is longer and the mass of the distribution is concentrated on the left side.

We estimated the skewness of our data for all seven experimental conditions and the aggregate condition using the functions for skewness estimation of the package ModeEst [40]. The result is shown in Table 2. Overall, the deviation from a perfectly symmetric distribution is very small in all cases (very far from ±0.5), with the most extreme values 0.061 and −0.064. Nevertheless, we note that the values that are farther away from zero are the ones from non-stimulated conditions, and the values that are closest to zero are the ones from stimulated conditions and the aggregate.

**Table 2.** Skewness estimates for the seven experimental conditions and the aggregate condition. The stimulated (S) conditions presented very low or negative values, whereas the non-stimulated (NS) conditions presented higher values. The aggregate condition displayed the positive value of skewness that was closest to zero, showing that the averaging process reduced the overall skewness

Condition	Skewness	Condition	Skewness
NS_PBMC_X4	0.046	S_PBMC_X4	−0.064
NS_PBMC_R5	0.031	S_PBMC_R5	−0.003
NS_CD4_R5	0.043	S_CD4_R5	0.016
NS_CD4_X4	0.061	AGGREGATE	0.008

### Exploratory data analysis

#### Pairwise two-sample Kolmogorov–Smirnov test

For the pairwise two-sample Kolmogorov–Smirnov test, the null hypothesis is that both empirical distributions were sampled from the same population. The results are shown in Table 3. The most important observation is that the seven experimental conditions were split into two groups according to the host cell state: (i) the stimulated conditions and (ii) the non-stimulated conditions. Two pairwise comparisons were marginally close to the rejection threshold: (1) NS_CD4_X4×S_PBMC_X4 and (2) S_CD4_R5×S_PBMC_X4.

**Table 3.** Pairwise two-sample Kolmogorov-Smirnov test with correction for multiple testing (number of tests K = 21). The significance level per test was set to *α* = 0.05, and using Bonferroni adjustment the total significance level was *α_B_* = 0.002. The table shows the *p*-values of all pairwise tests. Values equal or less than the significance level are marked in bold. Two pairs were marginally close to the significance level, namely NS_CD4_X4 × S_PBMC_X4 and S_CD4_R5 × S_PBMC_X4 (marked with an asterisk)

	NS_CD4_R5	NS_CD4_X4	NS_PBMC_R5	NS_PBMC_X4	S_CD4_R5	S_PBMC_R5	S_PBMC_X4
NS_CD4_R5	---	0.2	0.6	0.07	**5×10** ^**−6**^	**1×10** ^**−8**^	**2×10** ^**−5**^
NS_CD4_X4		---	0.03	0.02	**2×10** ^**−4**^	**2×10** ^**−6**^	*1×10 ^**−3**^
NS_PBMC_R5			---	0.1	**1×10** ^**−8**^	**1×10** ^**−9**^	**1×10** ^**−7**^
NS_PBMC_X4				---	**6×10** ^**−5**^	**2×10** ^**−6**^	**5×10** ^**−5**^
S_CD4_R5					---	0.5	*0.005
S_PBMC_R5						---	0.1
S_PBMC_X4							---

#### Impact of the exclusion of sites

We searched for patterns in the excluded dataset to reduce the possibilities for bias in our analysis. About 95% of all sites were considered for analysis, and 5% were discarded. The excluded sites were distributed throughout the genome, not indicating any preferential region of concentration.

In order to investigate how the excluded sites were distributed along the genome, we introduce the notion of *gap* generated by the exclusion. Given the *i*-th non-excluded site *s_i_*, we say that there is a *gap of length n* after *s_i_* if there are exactly *ℓ* (*ℓ* ⩾ 1) sites that were excluded between *s_i_* and the next non-excluded site *s_i_*_+1_; if the two non-excluded sites *s_i_* and *s_i_*_+1_ are consecutive sites in the genome, then we say that the gap length between them is *ℓ*=0.

The *distribution of gap lengths* is given by the proportions of gaps of length for each *ℓ*=0,1,…, *ℓ*_max_, along the genome. That is,


$$\Lambda (\ell )=\frac{\text{number of gaps of length }\ell }{\text{number of sites of the genome}}$$

Note that the number of gaps of length 0 is exactly the number of non-excluded sites. More generally, the number of gaps of length ℓ is the number of blocks of ℓ consecutive sites that were excluded.

**Fig. 5.**
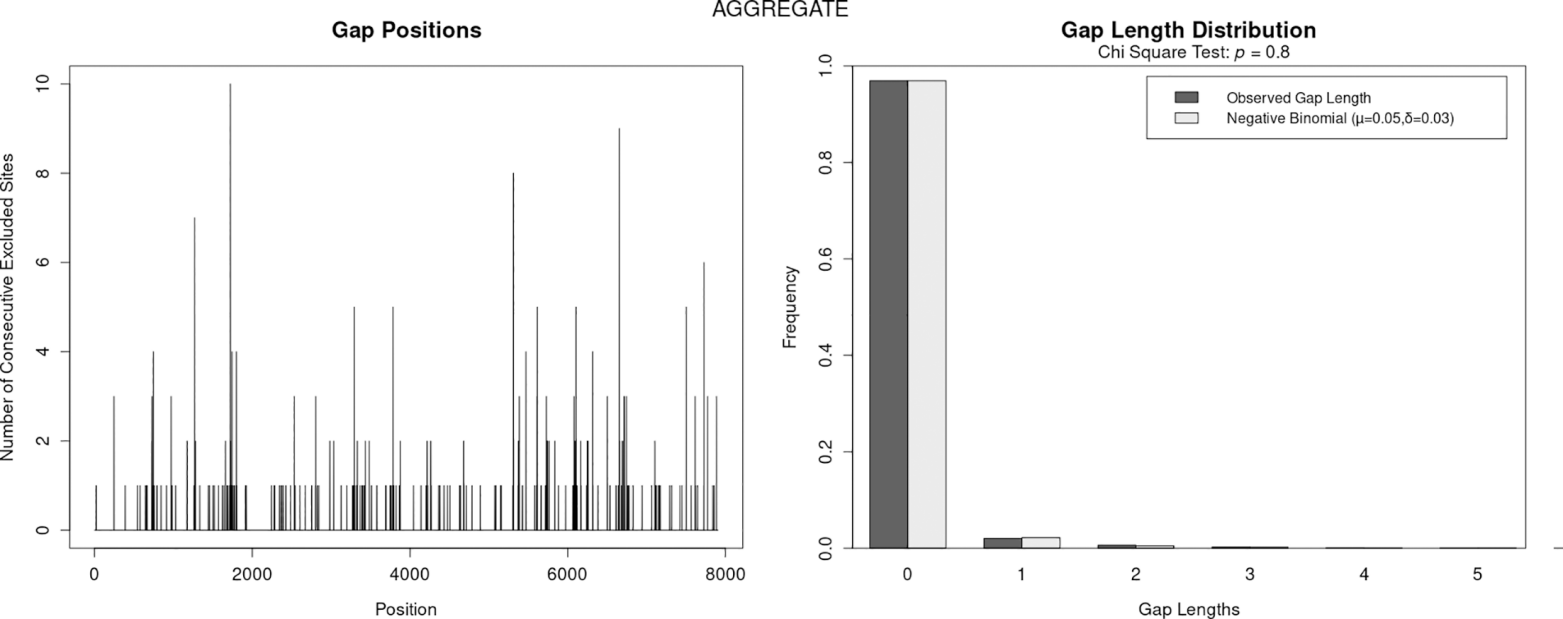
Gap positions and gap length distribution for the aggregate condition. (Left) Gap positions on the genome. The length of the bar is the number of consecutive sites that were excluded. The marks in the x-axis represent the number of non-excluded sites in the specific order and not their location in the genome. (Right) Distribution of observed gap lengths (dark grey) and expected negative binomial distribution (light grey) with parameters *μ*=0.05 (mean) and δ=0.03 (dispersion). For each length ℓ=0, 1,…, 10, the bars give the proportion of sites with length ℓ. Here, the x-axis shows the lengths up to ℓ=5.

In Fig. 5 (right), we plot the positions and the length of the gaps between the sites. The length of the vertical bar indicates the number of consecutive sites that were excluded right after the position where the bar is located. The plot displays an almost uniform distribution throughout the genome for the aggregate condition (see Fig. S3 of the Supplementary Material for all other conditions). The distribution of gap lengths is shown in Fig. 5 (left) for the aggregate condition. We performed chi-square tests for all conditions, showing that it follows a negative binomial distribution, which is the expected distribution for a random clustering of individual units [54].

Finally, we also verified if there were excluded sites that were recurrent in more than one condition. Considering the universe of analysed sites and conditions, we found that less than 1% of the sites appeared in two or more conditions.

### Scale invariance and information dimension

#### Genomic localization of the DMRs

Table 4 shows the summary statistics of the aggregate empirical DMRs for the whole genome, the genes and smaller fragments, up to a length of 50 nucleotides. The same result holds for all seven conditions (data not shown). Observe that there is a maintenance in the order of magnitude of the mode, the median and the mean, regardless of the length of the fragment, all the way to 50 nucleotides.

**Table 4.** Summary statistics of the empirical DMRs for the whole genome, genes, and smaller fragments, up to a length of 50 sites (aggregate condition). Min.: minimum value; Q_1_: first quartile; Q_3_: third quartile; Max.: maximum value; R.S.: proportion of retained sites

Genome / Gene	Min.	Q_1_	Mode	Median	Mean	Q_3_	Max.	R.S.
**HIV** (8,295 sites)	6.34×10^−5^	3.71×10^−4^	3.95×10^−4^	6.39×10^−4^	9.16×10^−4^	1.13×10^−3^	5.96×10^−3^	94%
***pol*** (3,012 sites)	6.39×10^−5^	3.67×10^−4^	3.96×10^−4^	6.24×10^−4^	9.11×10^−4^	1.12×10^−3^	5.90×10^−3^	95%
***env*** (2,571 sites)	6.37×10^−5^	3.54×10^−4^	3.53×10^−4^	6.23×10^−4^	8.89×10^−4^	1.10×10^−3^	5.88×10^−3^	92%
***gag*** (1,501 sites)	6.55×10^−5^	4.08×10^−4^	4.07×10^−4^	7.09×10^−4^	9.87×10^−4^	1.24×10^−3^	5.88×10^−3^	95%
***vif*** (579 sites)	6.98×10^−5^	4.19×10^−4^	4.62×10^−4^	6.77×10^−4^	9.61×10^−4^	1.16×10^−3^	5.54×10^−3^	97%
***rev*** (351 sites)	8.69×10^−5^	4.86×10^−4^	5.56×10^−4^	7.56×10^−4^	1.02×10^−3^	1.24×10^−3^	5.49×10^−3^	99%
***tat*** (306 sites)	9.13×10^−5^	4.22×10^−4^	4.90×10^−4^	6.72×10^−4^	9.64×10^−4^	1.17×10^−3^	5.43×10^−3^	98%
***vpr*** (292 sites)	7.42×10^−5^	3.93×10^−4^	4.03×10^−4^	6.61×10^−4^	9.23×10^−4^	1.12×10^−3^	5.11×10^−3^	97%
***nef*** (289 sites)	9.08×10^−5^	4.75×10^−4^	4.51×10^−4^	7.53×10^−4^	9.99×10^−4^	1.27×10^−3^	5.19×10^−3^	95%
***vpu*** (249 sites)	7.64×10^−5^	2.78×10^−4^	2.92×10^−4^	4.86×10^−4^	7.35×10^−4^	8.76×10^−4^	4.81×10^−3^	93%
***env_gp120*** **(incompl.)** (200 sites)	7.30×10^−5^	2.96×10^−4^	3.10×10^−4^	4.99×10^−4^	7.46×10^−3^	8.90×10^−3^	5.30×10^−3^	94%
***gag_p6*** (156 sites)	1.19×10^−4^	4.83×10^−4^	5.24×10^−4^	7.75×10^−4^	1.07×10^−3^	1.36×10^−3^	4.76×10^−3^	98%
***pol_int*** **(incompl.)** (100 sites)	7.66×10^−5^	3.58×10^−4^	4.18×10^−4^	5.57×10^−4^	8.81×10^−4^	1.03×10^−3^	4.86×10^−3^	93%
***gag_p6*** **(incompl.)** (50 sites)	1.73×10^−4^	5.28×10^−4^	5.68×10^−4^	8.92×10^−4^	1.20×10^−3^	1.66×10^−3^	4.18×10^−3^	99%

In order to verify if the trend shown in Table 4 is robust, we performed a simulation where we randomly picked fragments of different sizes and computed the associated DMR. More precisely, for a given fragment size *N*, we generated 1,000 random fragments of size *N*, from the whole genome, computed the log-DMR for each fragment and calculated the corresponding mean value. With this list of 1,000 mean values, we calculated the *range* (i.e. the difference ∆=max − min) and the *mean µ* (i.e., the mean of the mean values). We varied *N* in increasing order from 50 to 1,000 in steps of 25.

**Fig. 6.**
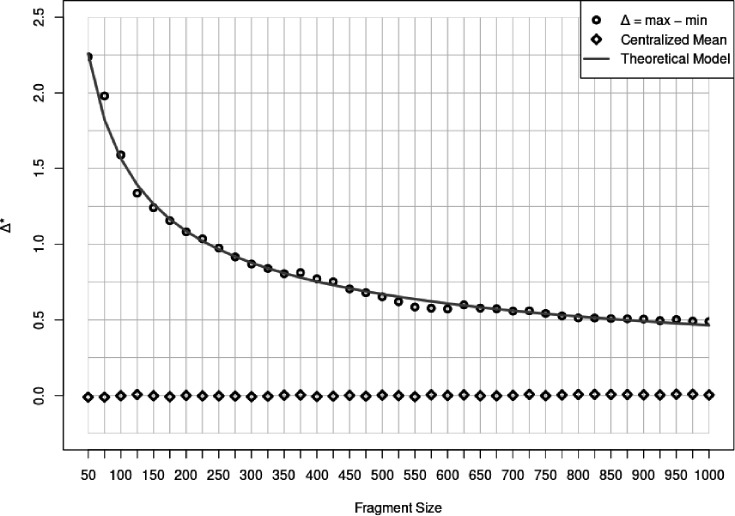
Simulation of random fragments of varying sizes *N* (aggregate condition). Range function ∆(*N*) (circle), centralized mean (over *N*) *μ*(*N*) − ⟨*μ*(*N*)⟩_*N*_ (diamonds), theoretical model for the range function, ∆^∗^(x)=1.5x^−0.5^, *p*-value<10^−16^, R-adjusted=0.99.


The result of this simulation is shown in Fig. 6 for the aggregate condition. There are three plots in this figure. The first plot (circle) is the range function ∆(*N*). The second plot (diamonds) is the ‘centralized mean’, given by *µ*(*N*) − ⟨*µ*(*N*)⟩*_N_*, the average of *µ*(*N*) over *N*. The third plot (solid line) is the theoretical model fitted to the data of the first plot, given by

$$\Delta^{*}(x)=1.5\;x^{-0.5}=\frac{1.5}{\sqrt{x}}$$

The function ∆^∗^(x) is a *power law*, that is, a function of the form $C\;x^{-\gamma }$, where *C* is a constant and *γ* is the *scaling exponent*. Although the parameter *N* is discrete – there is no such thing as a fragment of size 1/2 – the theoretical model allows us to interpolate the range function ∆(*N*) with a continuous function of a real variable *x*. This interpolation facilitates the analysis of the range function as we continuously vary the ‘fragment size’ from 1,000 to 50.

In our case, we are interested in the behaviour of the DMR as *N* → 0. Therefore, we can conclude that the range function ∆(*N*) increases as the fragment size *N* decreases, at the same rate as the reciprocal of the square root of *N*.

From Fig. 6, we can see that the estimate for the mean value itself barely changes as we vary *N*. Nevertheless, the divergence to infinity of ∆(*N*) as *N* → 0 shows that, as the size of the fragment approaches 0, the mutation rate estimate provided by the mean value becomes increasingly uncertain. There is not a sharp transition at any particular value, but a continuous increase following the power law scalingngng $x^{-\frac{1}{2}}$ .

The ‘power law scaling’ suggests that some statistical properties of the DMR (up to a certain limit) are independent of the length of the associated genomic fragment.

#### Estimation of information dimension

In order to apply the information dimension estimation, we recall that the genome length (i.e. the total number of sites after the application of exclusion criteria) is L≈7,800. Let *N* denote the number of fragments in the coarse-grained approximation. For example, if we take *N*=20, then the average fragment length is *ℓ*=*L*/*N*=390, and at this fragment length, the trend of the summary statistics is maintained (see Fig. 6). However, for *N*=50, 100 (*ℓ*=156, 78), the summary statistics start to diverge from the trend.

**Fig. 7.**
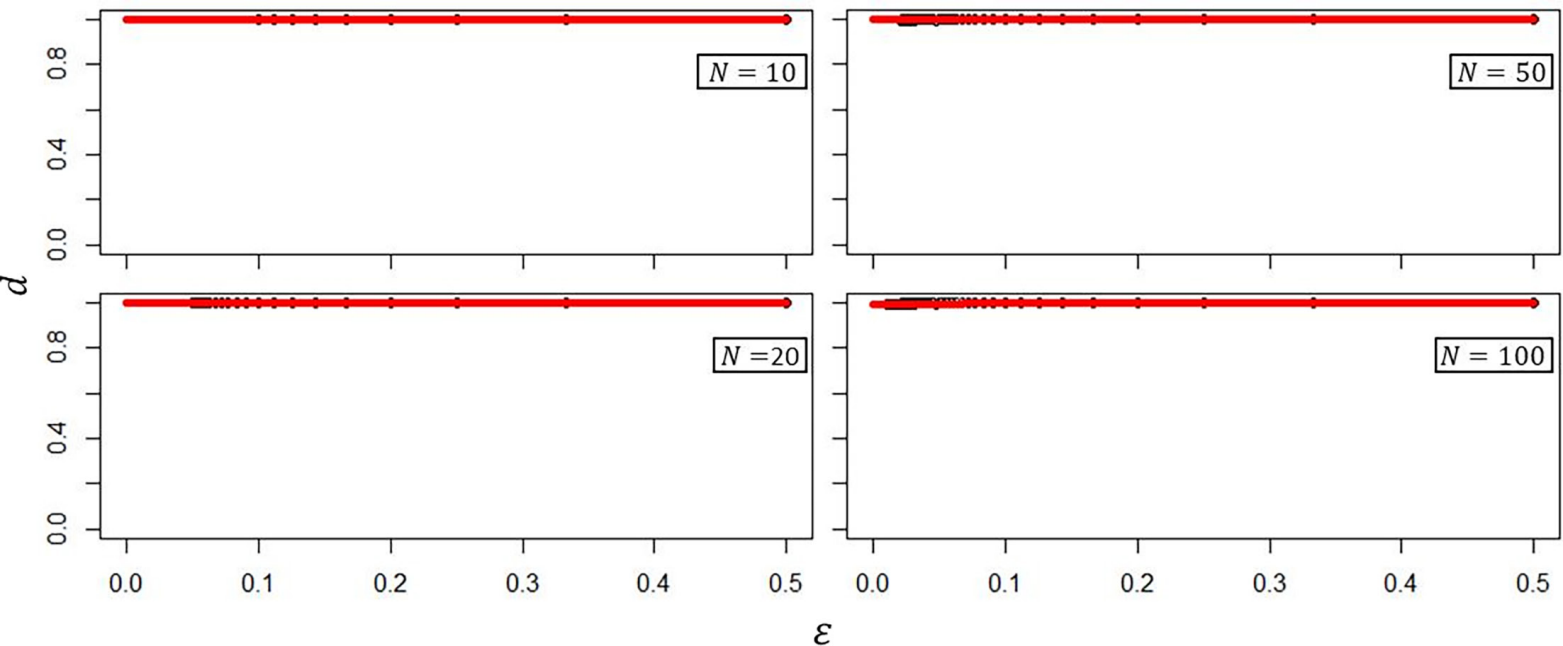
Estimation of Shannon information dimension *d*. Successive approximations of the information dimension (black dots), as function of the scaling parameter *ε*=1/*N* for *N*=2, 3,…, *N*_max_ with *N*_max_=10, 20, 50, 100 (aggregate condition). The estimate of the information dimension $\hat{d}$ is given by the red line obtained by linear regression. The slope estimate is 0.003 (*p*<0.001), and the intercept estimate is 0.9978 (*p*<10^−15^). Hence, the red line is parallel to the horizontal axis. The estimate of the information dimension is given by the intercept estimate: $\hat{d}$=0.9978±0.0004 (99% CI).

In Fig. 7, we show the successive approximations of the information dimension *d*, as a function of *ε*=1 */N* for *N*=2, 3, …, *N*_max_ with *N*_max_=10, 20, 50, 100, and its estimate (red line) (see Supplementary Material, Appendix C for details). The data used in the computation were from the aggregate condition. The seven experimental conditions give similar results (data not shown).

The information dimension is estimated to be $\hat{d}=0.9978\pm 0.0004$, i.e. it is very close to 1, and so, $\exp S(\varepsilon )\approx {(1/N)}^{-1}=N$. In other words, the average exponential Shannon information ‘per box’ of size ε=1/N scales as N. This type of scaling is expected for a *discrete uniform probability distribution* with N distinct outcomes, each having a probability 1/N. The discrete uniform distribution is the maximum entropy distribution among all discrete distributions that have N distinct outcomes. This implies that the complementary probabilities are distributed along genomic sufficiently long fragments as evenly as possible. Therefore, the associated DMR is independent of the fragment size, for sufficiently long fragments.

We performed two tests to check the robustness of the information dimension estimation. The first test is accomplished by rearranging the sites in such a way as to break the natural sequential order of the complementary probabilities along the genome. This test shows that the distribution of the complementary probabilities along the genome is crucial for the scale invariance to occur. Here, the crucial property of the distribution of $p_{comp}$ along the genome is that it is as evenly spread as possible. So much so that, when we disrupted this evenness by reordering, the scale invariance was lost.

**Fig. 8.**
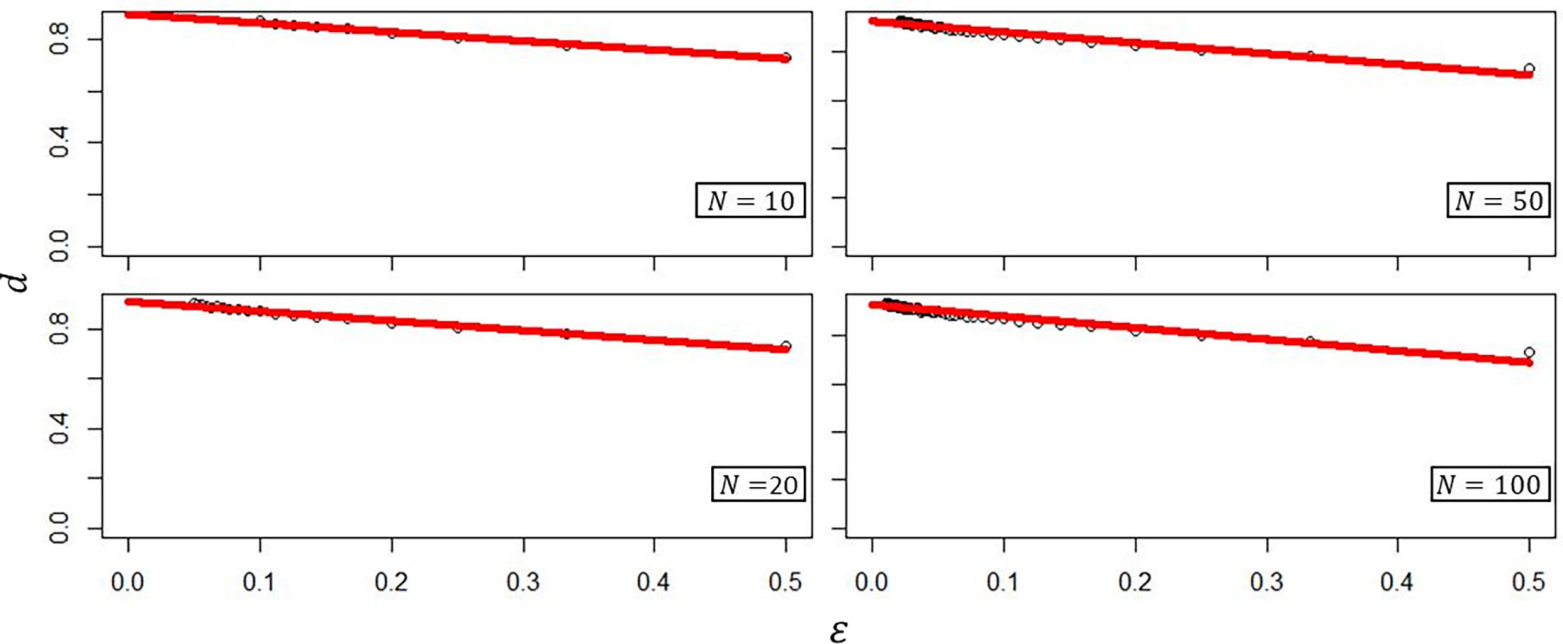
Shannon information dimension *d_ε_* as a function of the scaling parameter *ε*=1/*N* for *N*_max_=10, 20, 50, 100. The input data are the same as used in Fig. 7, but the sites were sorted in increasing order with respect to *p*_comp_. Here, the regression line (red line) is no longer horizontal.

In Fig. 8, we plot the same data as in Fig. 7, except that we sorted the sites in increasing order according to $p_{comp}$. The regression line used to estimate the information dimension is no longer horizontal. The slope is non-zero with high significance $\left(p§lt;10^{-15}\right)$ and hence, the estimate of the information dimension by the intercept of the regression line does not make sense anymore.

The second test consists of extending the notion of Shannon information dimension to the notion of q-deformed information dimension. This extension gives a continuous family $d_{q}$ of information dimensions, parameterized by a real parameter q, called *entropic order*, such that when q=1, we recover the Shannon information dimension: $d_{1}=d$. Different values of the entropic order q tell us different things about the corresponding system.

The advantage of defining a family of dimensions $d_{q}$ is that rather than picking one q to work with, we can consider all of them at once. When q≠1, the associated entropy is influenced by correlations or interactions between parts of the system. Thus, for a given system, one can similarly construct $d_{q}$ as it was done for the Shannon information dimension and plot $d_{q}$ against q (see Supplementary Material, Appendix C for details). This family of dimensions is called the *dimension profile* of the system and is quite informative.

Here, we consider only q≥0 and note that there are some special values of q for which $d_{q}$ is always the same for any system: $d_{0}=1$ and $d_{+\infty }=1$ (see Supplementary Material, Appendix A for the definition of $d_{+\infty }$). Because of these properties, it follows that $0\leq d_{q}\leq 1$ for all q≥0, i.e. $d_{q}$ is bound between 0 and 1. Furthermore, it follows that there is a parameter $q^{*}§amp;gt;0$ such that $d_{q^{*}}$ is minimal. In some cases, the parameter $q^{*}§amp;gt;0$ such that the dimension *d*_*q∗*_ is minimal is called the *natural entropic order* of the system.

**Fig. 9.**
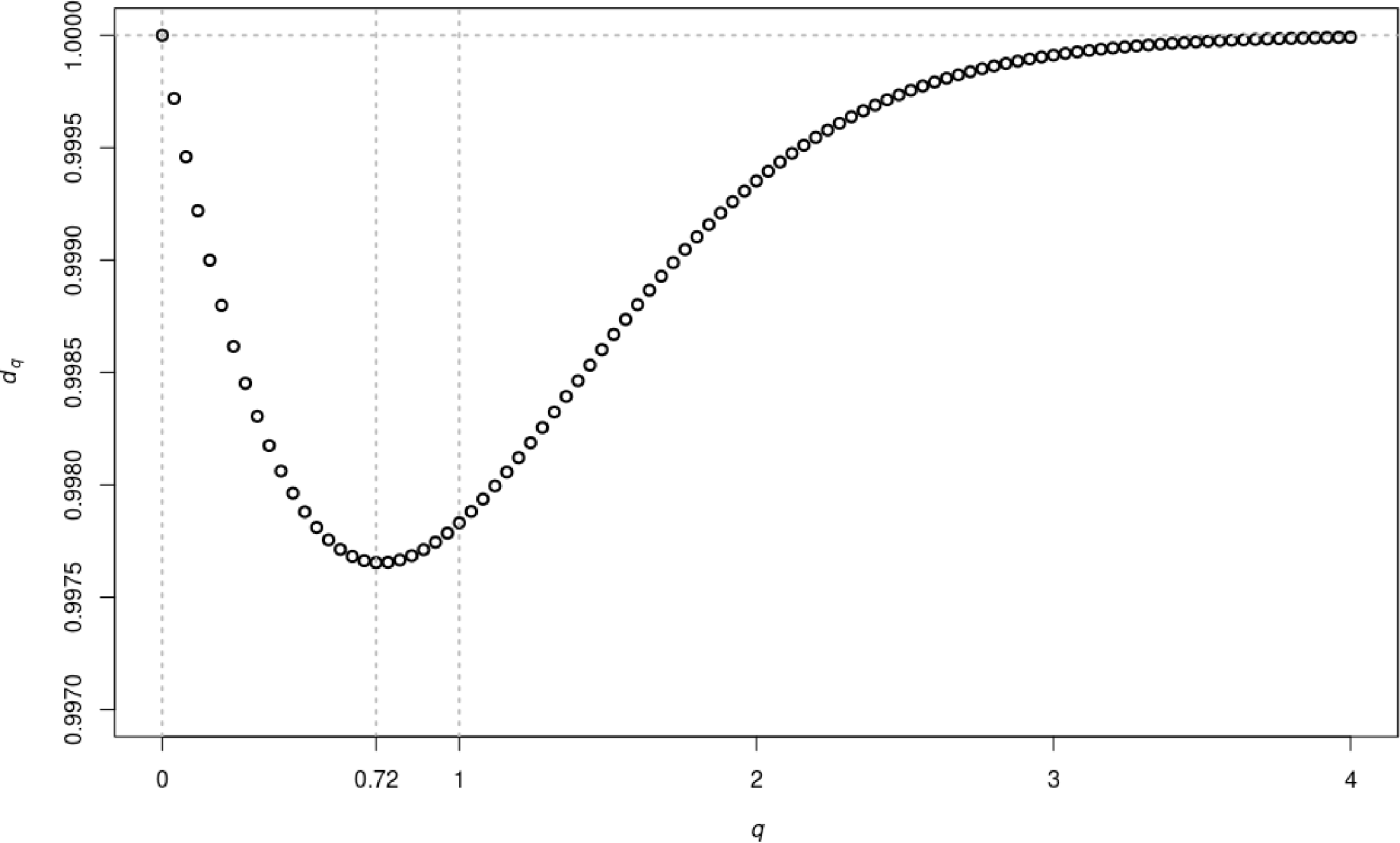
The *q*-logarithmic information dimension profile obtained from the same data as in Fig. 7 with *N*_max_=20. For *q*=1, one obtains the Shannon information dimension *d*=*d*_*1*_=0.9978 as shown in Fig. 7. The value of *q* that makes *d*_*q*_ minimal is *q*^*∗*^=0.72 with *d*_*q∗*_=0.9976.

In Fig. 9, we show the q-deformed information dimension profile obtained from the same data as in Fig. 7 with $N_{max}=20$. The fact that the Shannon information dimension $d=d_{1}$ is continuously interpolated by $d_{q}$ shows that, even though it is very close to 1, it is strictly smaller than 1. Moreover, the minimal dimension *d*_*q*^∗^_ is attained when *q*^∗^=0.72. Hence, the second test shows that the *q*-deformed information $d_{q}$ belongs to an interval $0.9976⩽d_{q}⩽1$, for all q⩾0. This means that the average exponential *q*-Rényi information scales as N, for all q. This corroborates the conclusion that the complementary probabilities are as evenly distributed as possible along sufficiently long genomic fragments. Furthermore, this conclusion holds independently of q, as well.

## Discussion

In this paper, we propose the use of the DMRs as a central concept for the study of mutation rates of RNA viruses. We showed that it can be estimated from sequencing data generated by an appropriate experimental design having a single replication round. The single replication round design allowed us to capture all mutational categories, including beneficial, neutral, deleterious and/or lethal, and yielded a substantial reduction of the bias from the effect of time on environmental selective pressure.

As mentioned in the introduction, there is a plethora of mutational rate values for several viruses, including HIV-1, HCV and influenza virus, in the literature. Usually, these estimates were obtained by distinct methodologies (e.g. mutation frequency, fluctuation tests) providing a single numerical value, a summarized mutational rate, often given by a mean or a median (often along with uncertainty quantification). The approach proposed here leverages the wide coverage and depth furnished by high-throughput sequencing technologies to estimate the DMR from sequencing data (see [20–23] for other approaches for viral mutation rate estimation using high-throughput sequencing).

Using the data generated by [19], we were able to successfully implement this approach by computing an empirical DMR for all seven experimental conditions considered by [19]. We also consider the average (geometric mean) of the seven experimental conditions to summarize the extensive data, giving rise to the aggregate condition. The averaging above is a natural operation to be performed with DMRs. Since the DMRs are expected to be well approximated by log-normal distributions, their geometric mean should also be well approximated by a log-normal distribution. Additionally, the averaging is expected to reduce the bias due to the differences in the experimental conditions (distinct host cell type and state, distinct envelope). Finally, we were able to show that all the empirical DMRs are indeed very well approximated by log-normal distributions. Granted that the empirical DMRs were well-characterized distributions, we proceeded further to study some of their basic properties. We computed some basic summary statistics of the empirical DMRs for all conditions and found a remarkable convergence of all central tendency measures: mode, median and mean (see Table 1).

An important assumption behind the notion of DMR is that of population heterogeneity in mutation rates [24]. In this framework, each individual samples the DMR for its mutation rate at a replication cycle. Then, the DMR only emerges at the large population level. Possibly, the variability of results from the literature is not an anomaly, but the result of insufficient sampling of the DMR. The ability to access the whole DMR may be hindered by several factors connected with experimental design. A viral population evolves into a dynamic equilibrium, where several events occur simultaneously. During this process, fully functional and/or defective viral particles are formed, selected and/or eliminated. Thus, non-functional and unselected events, which are part of the overall dynamics of the viral population, may be disregarded. Most likely, each work captured a small fraction of the DMR by sampling different regions of the distribution.

Even though our focus here is to develop an approach centred on the DMR, it is clear that it can be used to produce point and interval estimates of the mutation rates, for instance, using any one of the central tendency measures (mode, median and mean). The mode and the median are the most robust measures of central tendency – the mean is very sensitive to variation in the tail of the distribution. Another possibility is to use the median together with the IQR as a robust point/interval estimate for the mutation rate.

For example, from Table 1, we get that the IQR interval of the aggregate condition is $\left[3.7\times 10^{-4},1.1\times 10^{-3}\right]$ m/b/r. Now, this interval contains only a few of the several point estimates from the literature that were mentioned in the introduction (exactly the purified HIV-1 RT experiments). However, it is important to emphasize that such direct comparisons should be made very carefully. First, most point estimates from the early literature on mutation rate estimation, primarily concerning RNA viral populations, do not include uncertainty quantification (e.g. standard error or confidence interval). Nevertheless, more recent research using modern tools, such as recent estimates of SARS-CoV-2 mutation rates, for example, does present proper uncertainty quantification [12, 55]. Second, there are two main units used to measure mutation rates: (i) mutations per base per cell infection (m/b/c) and (ii) mutations per base per replication cycle (m/b/r). Consistently, the mutation rates per replication cycle are lower than those per cell infection. They are not the same, since in a cell infection several replication cycles may occur, that is, we can define the number of copying cycles per infected cell $r_{c}$, which is a positive integer, given by $\mu_{\left(m/b/c\right)}\;/\mu_{\left(m/b/r\right)}=r_{c}$. For HIV-1, we have that $r_{c}=1$, since the template RNA is destroyed after the retro-transcription, but for other viruses, it is possible to have $r_{c}§amp;gt;1$ [10]. Third, the environment where the host cells are located impacts the viral mutation rates [56, 57], as well as for bacteria mutation rates [58, 59]. Finally, we should mention that any apparent discrepancy is not surprising since it is expected that point/interval estimates obtained from the DMR tend to give higher values.

Despite the utility of the DMR to make point/interval estimates, it has much more potential to reflect the dynamics of a viral population than a single numerical value. By analysing how the DMR behaves under ‘localization’ from the genome level to gene level and then to gene sub-units, we identified an almost ‘scale invariance’ property of the DMR. This observation suggests that the probabilities of mutations spread evenly all over the genome, not displaying any preferred regions with concentration of high or low probabilities of mutation. As seen from the summary statistics (Table 4), there is a remarkable convergence, indicating the almost complete uniformity of randomness of mutation occurrence.

At first, it may seem that our result on the location randomness of mutation occurrence contradicts some well-established literature. For example, the HIV-1 *env* gene should display more mutations than other regions, considering that the envelope proteins are targets of neutralizing antibodies [60, 61]. However, the changes that are observed in these studies are in fact substitutions that were fixed along the lineages by the selection process, i.e. the substitution rate is affected by selection. Indeed, the *env* gene is strongly affected by the host’s immune system. If our experimental design considered more than one replication cycle, the viral population could be exposed to some selection mechanism, which could be biassed towards regions with more susceptibility, even in a cell culture. Within our setup of a single replication cycle, these biases towards specific regions are completely suppressed in practice.

An interesting question related to the emergence of scale invariance is the minimal length where the ‘scaling’ breaks down: when the fragment length is around the range 200 to 400 nucleotides, the summary statistics start to show some divergence from the initial trend, and the range function starts to blow up very fast. Viral genomes are the most compressed and highly optimized self-replicating genomes, presenting few or no redundancy at all [10, 62]. Of course, such extreme genome reduction is one of the intracellular obligatory entities’ strategies to accomplish the self-replication process. For example, the smaller known viruses belong to the *Circoviridae* family, presenting about 1.7 to 2.2 thousand nucleotides [63, 64]. The smallest unicellular organism is the *Mycoplasma genitalium* with a genome of about 580 kb [65, 66]. In 2016, [67] introduced the JCVI-syn3.0, a self-replicating cell with the smallest synthetic bacterial genome of 531 kbp. However, viroids can display genomes of 200 to 400 nucleotides [68], and about 80% of the human exons are about 200 nucleotides [69]. These last two facts, together with our analysis, suggest that the lower bound of ∼200 is the minimum length of a genomic fragment that can carry information on mutation rates.

The emergence of the scale invariance suggests two questions: (i) Is it possible to associate the presence of scale invariance of the DMR with some notion of information theory? (ii) What is the biological implication of this? Today, information theory is widely applied in biology, especially in sub-fields that deal with massive quantities of biological data, such as molecular biology, system biology, bioinformatics and computational biology [70–73], but also genetics and evolution theory [74, 75]. If information theory plays a role in the structure of DMR, then it should be through the notion of *information dimension*. Information dimension is a concept from information theory that quantifies the ‘complexity’ of a probability distribution, often revealing ‘fractal properties’, by measuring how information scales with some kind of resolution as an associated ‘state space’ is successively subdivided. It is a ‘fractal dimension’ for probability distributions, characterizing irregular structures and estimating the information gained from measurements [76–81]. In the last section, we have shown that the definition of the empirical DMR, coupled with the genomic localization procedure, defines a notion of resolution with respect to genomic fragments, and so one can associate an information dimension (based on Shannon entropy). As shown in Fig. 7, the information dimension is close to 1, independently of the fragment length. Therefore, the distribution of the complementary probabilities along a sufficiently long fragment is of maximum entropy (discrete uniform probability distribution). The biological implication of this conclusion is that the empirical DMR obtained from sequencing data is the same, regardless of whether the sequencing data covers the whole genome or a sufficiently long fragment. This is consistent with the observation that viral genomes exhibit high informational compression, with very few or no redundancy regions [10, 62].

The unusual robustness of the DMR shown in all our analyses leads us to a remarkable conclusion: the DMR constructed from the genome sequencing of HIV-1 is a ‘quasi-universal’ feature of the specific virus. In fact, as we mentioned before, the summary statistics of all seven conditions and the aggregate display sharp convergence in all statistics. The skewness estimation and the pairwise comparison using the two-sample Kolmogorov–Smirnov test concurrently split the conditions into two groups: (i) the stimulated conditions and (ii) the non-stimulated conditions. Therefore, apart from the subtle difference between stimulated and non-stimulated conditions, the empirical DMRs obtained from the seven conditions appeared to be essentially indistinguishable.

However, it is expected that the DMR should be virus-specific, i.e. it should depend on the size of the genome and the mode of replication of the virus. In fact, the so-called *Drake’s Rule* is the observation that within broad groups of organisms, the density of accumulated mutations per generation is roughly inversely proportional to genome size, which can vary by a few orders of magnitude [6, 7, 27–29, 82]. However, one should not expect the DMR to capture general characteristics of all viruses.

On a more speculative note, we would like to entertain possible explanations for the quasi-universality of the DMR. Recall that our experimental and analytical setup is designed to capture the rates of spontaneous mutations, which mainly occur due to errors made by DNA polymerases during replication. In a single-round infection event, with minimal selection pressure, the polymerase effects should be highlighted. Accordingly, any parameter associated with spontaneous mutations should be an intrinsic property of the replication machinery of the virus, independently of any exogenous influence.

When a DNA polymerase adds to the end (terminus) of a growing strand, sometimes a non-complementary unit can be added: this is often called a ‘misinsertion’ error. The resulting ‘terminal mispair’ can be corrected by a proofreading system if it exists; otherwise, it becomes an ‘internal mispair’, disrupting normal pairing and potentially leading to mutations. For example, in the *E. coli* polymerase III, misinsertions occur at a rate of 10^−4^ or 10^−5^ m/b/r for transition or transversion mispairs, respectively. The effectiveness of proofreading in this case is such that the rate of formation of internal mispairs is 50-fold to 100-fold lower than for terminal mispairs [83]. Nevertheless, most RNA viruses do not have any proofreading system (exceptions to this come from the order of *Nidovirales*, such as the coronaviruses). Hence, the expected mutation rate should be in the range [10^−4^, 10^−5^] m/b/r. Since most of point and interval estimates of mutation rates derived from the empirical DMR fall into this range, it is plausible to suppose that the empirical DMR really captured the spontaneous mutation rates.

Now one may ask: How does a misinsertion error originate? There are several non-exclusive possibilities. Errors may reflect ‘tautomeric shifts’ to alternative forms that favour non-canonical pairings: if the polymerase inserts a nucleotide while that nucleotide, or the template nucleotide, is in such an altered state, a non-canonical pairing is favoured. It has been determined that tautomerism occurs at a frequency $∼10^{-4}$ [84–86]. This is close to the spontaneous mutation rate reported here, but there is a problem: a tautomeric shift has to occur exactly at the same nucleotide that the polymerase is about to read. In any case, the joint probability of the simultaneous occurrence of these two events gives a (positive) lower bound to the spontaneous mutation rate, but it cannot exhaust the possibilities. For these two events, consider the genome has a length of ~10^−4^ nucleotides and that both events are independent, the probability of simultaneous occurrence of both is ~10^−8^. A second possible mechanism is related to non-canonical ‘wobble’ pairings of canonical bases, due to the flexibility of the DNA helix, which gives it the capability to accommodate slightly misshapen pairings [83]. A third possible mechanism of misinsertion could be caused by conformational shifts due to the quantum tunnelling of electrons. In particular, this means that ordinary replication errors may be subject to quantum indeterminacy [87]. It is very likely that in reality, these three mechanisms are at work simultaneously and non-independently (even in conjunction with others not mentioned above) to produce the spontaneous mutation rates that we observe.

The main purpose of this paper was to present the method for estimation of the DMR from sequencing data. Nonetheless, we addressed several topics that were not necessarily within this original scope. These considerations were included here with the intention of illustrating the types of analysis that can be performed with the DMR.

## Conclusions

In this paper, we have shown that it is possible to empirically estimate the DMRs from sequencing data. Furthermore, we were able to theoretically characterize the DMR as a log-normal distribution. By exploring the basic properties of the DMR, we have shown that it can give point and/or interval estimates of mutation rates that are reasonably close to estimates reported in the literature.

We have mentioned that the notion of DMR has been used in other papers [24, 25]. However, to the best of our knowledge, we seem to be the first to propose an estimation method for DMR, based on deep sequencing data coupled with an empirical Bayes estimation and a strategy to generate a single replication cycle formed from an initial clonal population of RNA virus. For that reason, we could not find any other experiment or sequencing dataset in the literature to which we could apply the methods of this paper.

The method proposed in this paper is based on two main ingredients: (i) a cell culture experimental design that provides exactly one replication cycle of a viral population and (ii) a method for estimation of mutation probabilities from high-throughput sequencing data. We are building on previous results on HIV-1 done by our group, where item (i) above was accomplished in [19] and item (ii) in [26].

Nevertheless, the method for DMR estimation can be, in principle, applied to any virus, as long as some conditions are fulfilled: (1) as mentioned before, the viral population must undergo a single replication cycle. In the situation where it is not possible to ensure a single replication cycle, but only single-cell infections, one should employ the appropriate unit of measurement; (2) one must have genome samples before (at least one) and after (as many as desired) a single replication cycle, and all samples must be sequenced. For instance, in our case, the genome sample before the replication cycle was the plasmidial clone genome, called the BC condition; (3) with the (at least) two sequencing data mentioned above (before and after one replication cycle), one can use the methods in [26] to obtain the probabilities of the four bases (p_A, p_T, p_G and p_C, with p_A+p_T+p_G+p_C=1). Granted these three conditions, the construction of the empirical DMR follows as described before. The message here is that spontaneous mutation rate must be viewed as a random quantity, not a fixed deterministic constant. In other words, even in point estimation, the stochasticity does not come only from the measurement error, but it is an intrinsic property of population dynamics.

These findings are expected to open new avenues in the analysis of mutation rates. Details of data from *in vitro* experiments are expected to be more readily available, so that *in silico* experiments can be used as complementary experiments and vice versa.

## Supplementary material

10.1099/mic.0.001717 Supplementary Material 1.

## Abbreviations

BC: baseline control
CD4: CD4+ T lymphocytes
DMRs: distribution of mutation rates
HCV: hepatitis C virus
IQR: interquartile range
m/b/c: mutations/base/cell infection
m/b/r: mutations/base/replication cycle
NS: Non-stimulated condition
PBMC: Peripheral Blood Mononuclear Cells
R5: CCR5 chemokine receptor
S: Stimulated condition
X4: CXCR4 chemokine receptor
