# Supplementary material for: Enlarging viral mutation estimation: a view from the distribution of mutation rates
Source: Microbiology (Reading). 2026 Aug 12;172(8):001717. doi: 10.1099/mic.0.001717 (PMC13465770; doi:10.1099/mic.0.001717)
Supplement: Supplementary file 1 [file mic-172-01717-s001.pdf]

Supplementary Material to "Enlarging viral mutation estimation: a view from the distribution of mutation rates"

Taimá Naomi Furuyama[1,†] Isabel Maria Vicente Guedes de Carvalho[1,†,*]
Luiz Mário Ramos Janini[2,‡] Fernando Antoneli[3,‡]

April 29, 2026

A Estimation of the Probability Distributions of Nucleic Bases

In this appendix we provide an update and adaptation to other sequencing technologies of the method of estimation of nucleic bases probabilities from [17].

It is important to ensure that it is possible to carry out same analysis using other sequencing technologies. Because the one used in the original paper [17], the SOLiD™ technology, has been discontinued.

We assume that there are two sequencing experiments: the 'baseline' and an one 'experimental condition', following the experimental protocol described in [8], with the exclusion of the sequencing part. Instead, we assume that the sequencing was performed according to the specification of the manufacturer. The resulting data should be stored in the form of FASTQ files.

A.1 Read Mapping / Alignment

In [17] the read mapping / alignment step was performed in an unusual way due to the specificity of the SOLiD™ technology. With the current sequencing technologies there is standard way to carry out the read mapping / alignment step. This standard method amounts to computing a SAM / BAM file (Sequence / Binary Alignment Map).

A.2 Nucleic Base Counting

Now we assume that there two sets of BAM files: the 'baseline' and the 'experimental condition'.

The first step is to extract the raw nucleic bases counts for each site from the BAM files. This can be done with basic Bioinformatics software, such as, SAMtools/BCFtools [1, 5] or IGVtools [11, 12]. The resulting files contains a table with at least 4 columns, corresponding to the counts of the 4 nucleic bases (A, C, G, T) – usually there are more columns including: deletion (DEL), insertion (INS), unknown (N), average quality scores, etc. We will not consider this additional information at the moment. Then, each row corresponds to a sequenced site.

[1]Lab. Virologia, Instituto Butantan, São Paulo, SP, Brazil. (taimafuruyama@gmail.com, isabel.mello@butantan.gov.br)
[2]Departamento de Microbiologia, Imunologia e Parasitologia, Departamento de Medicina, Laboratório de Retrovirologia, Universidade Federal de São Paulo, São Paulo, SP, Brazil. janini@unifesp.br
[3]Centro de Bioinformática Médica, Universidade Federal de São Paulo, São Paulo, SP, Brazil. fernando.antoneli@unifesp.br
[†]These authors share the first authorship.
[‡]These authors share the senior authorship.
[*]Corresponding author.

A.3 Base Probability Distribution Estimation

The *base probability distribution*, at a given site, is the *multinomial probability distribution* for the occurrence of one of the 4 bases at that given site. It is a list $(p_{\rm A}, p_{\rm C}, p_{\rm G}, p_{\rm T})$ of 4 real numbers between 0 and 1, with $p_{\rm A} + p_{\rm C} + p_{\rm G} + p_{\rm T} = 1$.

In order to estimate the base probabilities from the raw counts we employ a *parametric empirical Bayes* estimation method, that is, the *prior probability distribution* is estimated from the data using a maximum likelihood estimation procedure [7]. This estimation procedure in a Bayesian setting is done through the use of the *Dirichlet distributions* associated to the *multinomial distributions* (see [9] for details). In our case, the associated Dirichlet distributions are parametrized by a vector with positive real entries $\boldsymbol{\alpha} = (\alpha_{\rm A}, \alpha_{\rm C}, \alpha_{\rm G}, \alpha_{\rm T})$, called *hyper-parameters* or *pseudo-counts*. These hyper-parameters are estimated from the raw counts and the multinomial probabilities are given by

$$p_J = \frac{\alpha_J}{\sum_J \alpha_J}$$

for $J \in \{\rm A, C, G, T\}$. The number $\alpha_0 = \sum_J \alpha_J$ is called *concentration parameter*.

Therefore, the method consists in estimating the vector pseudo-counts $\boldsymbol{\alpha} = (\alpha_{\rm A}, \alpha_{\rm C}, \alpha_{\rm G}, \alpha_{\rm T})$ from the actual raw counts $(c_{\rm A}, c_{\rm C}, c_{\rm G}, c_{\rm T})$ for each site. The method is divided into three stages (here K is the number of sites in the genome):

(1) In the first step, we use the 'baseline' data $\{(c^0_{\rm A}, c^0_{\rm C}, c^0_{\rm G}, c^0_{\rm T})_i : i = 1, \ldots, K\}$ to estimate the *prior hyper-parameters* $\{(\alpha^0_{\rm A}, \alpha^0_{\rm C}, \alpha^0_{\rm G}, \alpha^0_{\rm T})_i : i = 1, \ldots, K\}$. Here, the superscript 0 indicates 'prior'.

(2) In the second step, we use the 'experimental condition' data $\{(c^1_{\rm A}, c^1_{\rm C}, c^1_{\rm G}, c^1_{\rm T})_i : i = 1, \ldots, K\}$, together with the prior hyper-parameters $\{(\alpha^0_{\rm A}, \alpha^0_{\rm C}, \alpha^0_{\rm G}, \alpha^0_{\rm T})_i : i = 1, \ldots, K\}$, to estimate, via *Bayes formula*, the *unadjusted posterior hyper-parameters*.

(3) Finally, in the third step we use the prior hyper-parameters together sequencing error rate to obtain the *adjusted posterior hyper-parameters* $\{(\alpha^1_{\rm A}, \alpha^1_{\rm C}, \alpha^1_{\rm G}, \alpha^1_{\rm T})_i : i = 1, \ldots, K\}$. Here, the superscript 1 indicates 'adjusted posterior'.

The first step amounts to the use of an iteration of a fixed-point scheme that converges to the unique maximum of the log-likelihood function (see [17]). There are several options, such as the classical *Newton-Raphson*, the *Expectation-Maximization*, etc. [6, 9, 15]. Briefly speaking, the method consists in iterating an equation of the form

$$\boldsymbol{\alpha}^{\rm new} = \Phi(\boldsymbol{\alpha}^{\rm old}, \log \bar{\boldsymbol{p}})$$

where Φ is an appropriate mapping, $\log \bar{\boldsymbol{p}} = (\log \bar{p}_{\rm A}, \log \bar{p}_{\rm C}, \log \bar{p}_{\rm G}, \log \bar{p}_{\rm T})$ is a vector of 'log-frequencies', called *sufficient statistics*, and the first $\boldsymbol{\alpha}^{\rm old}$ is a vector of hyper-parameters used to start the iteration scheme, called *initialization vector*.

The main difficulty in applying this method to genomic data is due to the *sparsity* of the data. That is, the the majority of raw counts $(c_{\rm A}, c_{\rm C}, c_{\rm G}, c_{\rm T})$, have one or more entries c_J equal to 0. This is a serious obstruction to the estimation of the hyper-parameters because the log-likelihood function goes to infinity when some α_J goes to zero. Hence, the presence of zero raw counts makes the the method very unstable or even divergent. In order to fix this problem we have to resort to a procedure of *pseudo-sampling with noise-smoothing*. This procedure consists of creating a pseudo-sample from the the raw counts with the addition of a small random noise and then use this pseudo-sample to compute the sufficient statistics.

Given raw counts $(c_{\rm A}, c_{\rm C}, c_{\rm G}, c_{\rm T})$, let $C = c_{\rm A} + c_{\rm C} + c_{\rm G} + c_{\rm T}$ be the *absolute coverage depth* at the site. We compute a matrix S of *pseudo-samples*, with 4 columns and $N = C + 4$ rows

$$S = \begin{bmatrix} s_{1,\rm A} & s_{1,\rm C} & s_{1,\rm G} & s_{1,\rm T} \\ \vdots & \vdots & \vdots & \vdots \\ s_{N,\rm A} & s_{N,\rm C} & s_{N,\rm G} & s_{N,\rm T} \end{bmatrix}$$

such that each row $(s_{i,\rm A}, s_{i,\rm C}, s_{i,\rm G}, s_{i,\rm T})$ of S is a random sample satisfying

(1) $s_{i,J}$ is a non-negative integer for all i and $J \in \{\mathrm{A}, \mathrm{C}, \mathrm{G}, \mathrm{T}\}$,

(2) $(s_{i,\mathrm{A}}, s_{i,\mathrm{C}}, s_{i,\mathrm{G}}, s_{i,\mathrm{T}}) \approx (c_\mathrm{A}, c_\mathrm{C}, c_\mathrm{G}, c_\mathrm{T})$, for all i,

(3) $\dfrac{s_{i,J} + \eta}{s_i + 4\eta} \approx \dfrac{c_J + 1}{C + 4}$, for all i and $J \in \{\mathrm{A}, \mathrm{C}, \mathrm{G}, \mathrm{T}\}$.

Here, $s_i = s_{i,1} + s_{i,2} + s_{i,3} + s_{i,4}$ and η is a small fixed number, called *smoothing noise*. Finally, define the sufficient statistics $\log \bar{p}_J$, for $J \in \{\mathrm{A}, \mathrm{C}, \mathrm{G}, \mathrm{T}\}$, as the average of 'log-frequencies',

$$\log \bar{p}_J = \frac{1}{N} \sum_{i=1}^{N} \ln \frac{s_{i,J} + \eta}{s_i + 4\eta}$$

for $J \in \{\mathrm{A}, \mathrm{C}, \mathrm{G}, \mathrm{T}\}$. It is easy to perform the random sampling with the conditions described above in all statistical / mathematical software, such as R, Matlab and Python.

As for the initialization vector $\boldsymbol{\alpha}^{\mathrm{old}}$, the most recommended choice, called *Ronning's initialization step* [9], is defined by setting $\alpha_{\min} = \min\{s_{j,k}\}$ and

$$\boldsymbol{\alpha}^{\mathrm{old}} = (\alpha_{\min}, \ldots, \alpha_{\min})$$

In our case, it always happens that $\alpha_{\min} = \eta$.

With these data, one can run the iteration scheme above and obtain the maximum likelihood estimate of the hyper-parameters as its fixed point

$$\hat{\boldsymbol{\alpha}} = \Phi(\hat{\boldsymbol{\alpha}}, \log \bar{\boldsymbol{p}})$$

In fact, $\hat{\boldsymbol{\alpha}}$ is a finite precision approximation to the true fixed. In any case, we use this solution as the prior hyperparameter: $\alpha_J^0 = \hat{\alpha}_J$, for $J \in \{\mathrm{A}, \mathrm{C}, \mathrm{G}, \mathrm{T}\}$. There are efficient implementations of the maximum likelihood estimation of Dirichlet hyper-parameters in R, [13], Matlab [6] and Python [15].

The second step is the application of *Bayes formula* to obtain the (unadjusted) posterior hyper-parameters. Given that the Dirichlet distribution is the *conjugate prior* of the multinomial distribution [9], *Bayes formula* at the level of hyper-parameters is deceptively simple. If $(c_\mathrm{A}^1, c_\mathrm{C}^1, c_\mathrm{G}^1, c_\mathrm{T}^1)$ is a list of raw counts at a given site and $(\alpha_\mathrm{A}^0, \alpha_\mathrm{C}^0, \alpha_\mathrm{G}^0, \alpha_\mathrm{T}^0)$ is the list of prior hyper-parameters at the same site, then the list of unadjusted posterior hyper-parameters $(\alpha_\mathrm{A}^*, \alpha_\mathrm{C}^*, \alpha_\mathrm{G}^*, \alpha_\mathrm{T}^*)$ is given by

$$\alpha_J^* = \alpha_J^0 + c_J^1$$

for $J \in \{\mathrm{A}, \mathrm{C}, \mathrm{G}, \mathrm{T}\}$.

The third step consists in computing a correction factor based on the sequencing error rate of the equipment and use this to define the adjusted posterior hyper-parameters. Let us denote the sequencing error rate by ε – in the case of the SOLiD™ sequencing technology used in [8] we had $\varepsilon = 0.0006$. Consider the *prior complementary probability* at a given site $p_{\mathrm{comp}}^0 = 1 - \max\{p_\mathrm{A}^0, p_\mathrm{C}^0, p_\mathrm{G}^0, p_\mathrm{T}^0\}$, where $p_J^0 = \alpha_J^0 / \alpha_0^0$. Then the *correction factor*, at the given site, is $\varepsilon \times p_{\mathrm{comp}}^0$. Thus, the adjusted posterior hyper-parameter, at the given site is,

$$\alpha_J^1 = |\alpha_J^* - \varepsilon \times p_{\mathrm{comp}}^0 \times \alpha_0^*| = |\alpha_J^* - \varepsilon \times p_{\mathrm{comp}}^0 \times (\textstyle\sum_J \alpha_J^*)|$$

for $J \in \{\mathrm{A}, \mathrm{C}, \mathrm{G}, \mathrm{T}\}$. Here, the modulus $|\cdot|$ is to ensure that α_J^1 is positive.

It is important to note that in order to successfully apply this method the *mean depth coverage* (i.e. the total number of bases sequenced divided by the genome size) of the sequencing should be reasonably high. Usually, A mean coverage depth of 100× to 300× is generally sufficient for identifying viruses and generating consensus sequences, but to identify minor variants or quasispecies, with confidence, high coverage depths of above 1000× are recommended. For instance, in [8, Tab. 3] the mean depth coverage obtained with the SOLiD™ sequencing technology was $\sim 50,000\times$. Illumina™ sequencing technology typically allows for high coverage in samples with good viral titers, with reported average depths often exceeding 1000× in validated protocols. This should be sufficient to apply the method present here.

In Table 1 we show the error rates of several Illumina™ sequencing platforms currently available, recently obtained by [16]). The values for ε can be obtained from the column 'Error rate (%) / Median' by dividing the appropriate value by 100.

		Error rate (%)	
Platform	Number of samples	Median	Standard deviation
MiSeq	212	0.473	0.938
MiniSeq	40	0.613	0.459
NextSeq 500	160	0.429	0.827
NextSeq 550	171	0.593	0.435
HiSeq 2500	141	0.112	0.544
NovaSeq 6000	239	0.109	0.350
HiSeq X Ten	163	0.087	0.126

Table 1: Summary statistics for the observed error rates (%) of samples from each ILLUMINA™ sequencing platform (extracted from [16]).

B Nucleotide Composition, Mutation Spectrum and Transition to Transversion Ratio

In this appendix we perform a comparative analysis (nucleotide composition, mutation spectrum, transversion and transition rates and and transition to transversion ratio) using the full set of probability distributions of nucleotides per site from [8]. This can be used as a 'sanity check' on results of the estimation procedure explained in the previous appendix.

Let $\{(p_{\mathrm{A}}, p_{\mathrm{C}}, p_{\mathrm{G}}, p_{\mathrm{T}})_i : i = 1, \ldots, K\}$ be the set of probability distributions of nucleotides per site of a given experimental condition, where K is the total number of sites. For each nucleotide $B \in \{\mathrm{A}, \mathrm{C}, \mathrm{G}, \mathrm{T}\}$ consider the following set

$$B_{\uplus} = \{i : \arg\max\{p_{\mathrm{A},i}, p_{\mathrm{C},i}, p_{\mathrm{G},i}, p_{\mathrm{T},i}\} = B\}$$

Alternatively, if we consider the *complementary probability*, at the i-th site

$$p_{\mathrm{comp},i} = 1 - \max\{p_{\mathrm{A},i}, p_{\mathrm{C},i}, p_{\mathrm{G},i}, p_{\mathrm{T},i}\}$$

then

$$B_{\uplus} = \{i : p_{B,i} = 1 - p_{\mathrm{comp},i} = \max\{p_{\mathrm{A},i}, p_{\mathrm{C},i}, p_{\mathrm{G},i}, p_{\mathrm{T},i}\}\}$$

That is, $B_{\uplus}$ is the set of sites such that, B is the most prevalent base, namely, p_B is the highest among the 4 probabilities. It is clear that $B_{\uplus} \cap B'_{\uplus} = \varnothing$ if and only if $B_{\uplus} \neq B_{\uplus'}$ and the union of the sets $B_{\uplus}$ is the set of all sites. Thus, if we denote by $\#B$ the size of the set B, we have that

$$K = \#\mathrm{A}_{\uplus} + \#\mathrm{C}_{\uplus} + \#\mathrm{G}_{\uplus} + \#\mathrm{T}_{\uplus}$$

The *nucleotide composition* of the genome is the proportion of each nucleic base in the set of all nucleic bases of the genome. Define

$$B\% = \frac{\#B_{\uplus}}{K} \times 100\%$$

for each $B \in \{\mathrm{A}, \mathrm{C}, \mathrm{G}, \mathrm{T}\}$. Then, $B\%$ is a good approximation of the actual proportions nucleic bases.

Now, to define the *mutation spectrum* we consider the following quantities. Let B, B' denote to nucleic bases with $B' \neq B$ We denote the rate of change $B' \to B$ by B'/B. Let $\mathcal{N} = \sum_{i=1}^{K} p_{\mathrm{comp,i}}$ then

$$B'/B = \frac{1}{\mathcal{N}} \sum_{i \in B_{\uplus}} p_{B',i} \times 100\%$$

for $B', B \in \{\mathrm{A}, \mathrm{C}, \mathrm{G}, \mathrm{T}\}$, with $B' \neq B$. Note that there are 12 possibilities for the quantities B'/B when $B', B \in \{\mathrm{A}, \mathrm{C}, \mathrm{G}, \mathrm{T}\}$, with $B' \neq B$ and $\sum_{B' \neq B} B'/B = 100\%$.

With this information we can compute the transition and transversion rates. The *transition rate* (TS) is

$$\text{TS} = (\text{A/G} + \text{T/C} + \text{G/A} + \text{C/T}) \times 100\%$$

and the *transversion rate* (TV) is

$$\text{TV} = 100\% - \text{TS}$$

The *transversion to transition ratio* is give by

$$\text{TS/TV} = \frac{\text{A/G} + \text{T/C} + \text{G/A} + \text{C/T}}{1 - (\text{A/G} + \text{T/C} + \text{G/A} + \text{C/T})}$$

Table 2 shows all the quantities described above for all experimental conditions. The results are consistent with the literature. In fact, HIV-1 is highly rich in A, less rich in G, and markedly deficient in G. This A-bias is associated with a strong guanine-to-adenine (G $\to$ A) mutation bias on the plus strand. Additionally, it exhibits a strong bias toward transitions over transversions, resulting in a *transition to transversion ratio* TS/TV, often reported around 1.4 or higher.

	NS_CD4_R5	NS_CD4_X4	NS_PBMC_R5	NS_PBMC_X4	S_CD4_R5	S_PBMC_R5	S_PBMC_X4
A%	37.01	37.09	37.00	37.02	37.01	37.05	37.03
T%	22.29	22.30	22.12	22.28	22.30	22.79	22.27
C%	17.07	17.20	17.38	17.17	17.10	17.01	17.08
G%	23.63	23.41	23.50	23.53	23.59	23.10	23.62
C/A	18.33	13.65	13.52	11.47	13.80	12.70	11.80
**G/A**	28.90	29.44	30.66	28.32	29.58	31.71	29.78
T/A	3.36	4.11	2.81	3.11	4.13	3.19	3.76
A/T	5.83	7.90	7.24	7.17	7.53	7.22	6.20
**C/T**	14.50	11.16	10.25	13.97	10.38	10.56	12.73
G/T	5.72	3.46	4.24	3.91	3.57	4.12	3.85
A/C	4.96	2.17	2.75	4.84	2.76	2.34	3.56
G/C	1.16	1.45	0.92	1.23	1.10	1.20	0.67
**T/C**	9.54	7.42	7.76	8.27	7.68	6.93	9.65
**A/G**	11.90	13.86	14.31	13.34	14.15	14.87	13.39
C/G	0.81	2.83	2.13	1.22	2.70	2.16	1.25
T/G	1.99	2.55	3.40	3.13	2.51	2.90	3.35
TS	64.84	61.88	62.98	63.90	61.90	64.15	65.56
TV	35.16	38.12	37.02	36.10	38.10	35.85	34.44
TS/TV	1.84	1.62	1.70	1.77	1.62	1.79	1.90

Table 2: Nucleotide composition $B\%$, mutation spectrum B'/B, transversion (TS) and transition rates (TV) and transition to transversion ratio (TS/TV) of the 7 experimental conditions, computed from the estimated probability distributions per site. Compare with the nucleotide composition estimation from the raw data in [8, Tab. 2].

C Entropy and Information Dimension

The *Shannon entropy* of a finite probability distribution $P = \{p_i : i = 1, \ldots, k\}$, with $0 < p_i < 1$ and $\sum_{i=0}^{k} p_i = 1$, is given by [14]:

$$S = -\sum_{i=1}^{k} p_i \ln p_i \tag{C.1}$$

In Shannon's original information-theoretic formulation, the logarithm was given in base 2 ($\ln_2$), so that the Shannon entropy represents the expected number of yes/no questions necessary to determine an object's classification. Since then, it has become more common to use either the natural (base e) logarithm (ln or ln) or the base 10 logarithm ($\ln_{10}$). In this paper, we will always use the natural logarithm, denoted by 'ln' and its corresponding exponential function denoted by 'exp'.

A coarse-grained approximation of a system is given by a partition of its state space X. A *partition* $\mathcal{A} = \{A_j : j = 1, \ldots, N\}$ of a set X is a collection of nonempty, non-intersecting measurable sets A_j that cover X. Thus, there is an element of the partition corresponding to every possible outcome of a measurement of the system [2]. Assume that a probability distribution $P_{\mathcal{A}} = \{p_j = p(A_j) : j = 1, \ldots, N\}$ is attached to the partition $\mathcal{A}$ – here p is a function that associates a real number in $(0, 1)$ to each set A_j, such that $\sum_{j=0}^{N} p(A_j) = 1$. Then we define the entropy of the partition $\mathcal{A}$ by

$$S_{\mathcal{A}} = -\sum_{j=1}^{N} p_j \ln p_j \tag{C.2}$$

Let $\mathcal{A}_N$ and $\mathcal{A}_M$ be finite partitions of the state space with $N < M$. We say that $\mathcal{A}_M$ is a *refinement* of $\mathcal{A}_N$ and write $\mathcal{A}_N \leq \mathcal{A}_M$ if every set in $\mathcal{A}_N$ is a union of sets in $\mathcal{A}_M$. Given sequence of partitions $\mathcal{A}_1 \leq \mathcal{A}_2 \leq \ldots \leq \mathcal{A}_N$ of the state space X, one can consider a sequence of probability distributions $P_{\mathcal{A}_N}$ and the corresponding sequence of entropies $S_{\mathcal{A}_N}$. The *information dimension* of the system is defined by

$$d = \lim_{N \to +\infty} \frac{-\sum_{j=1}^{N} p_j \ln p_j}{\ln N} = \lim_{N \to +\infty} \frac{S_{\mathcal{A}_N}}{\ln N} \tag{C.3}$$

Note that the distributions $P_{\mathcal{A}_N}$ are univariate, hence it can be shown that $0 \leq d \leq 1$ [10].

The intuition behind this definition is the following. Suppose that we have a notion of "size" of the sets A_j of a partition $\mathcal{A}_N$ and that each set has approximately the same "size", $\varepsilon \approx 1/N$. In this setting, each set A_j is called a *box*, and thus ε is the "box size" parameter. Then we can write

$$d \approx \frac{S(\varepsilon)}{-\ln \varepsilon} \qquad \text{for} \quad \varepsilon \to 0^+ \tag{C.4}$$

where $S(\varepsilon) = S_{\mathcal{A}_N}$ is the average information per box. Hence, the information dimension d is the exponent of the box size scaling of the average information needed to identify an occupied box, that is

$$\exp S(\varepsilon) \approx \varepsilon^{-d} \qquad \text{for} \quad \varepsilon \to 0^+ \tag{C.5}$$

The exponential of the Shannon entropy $\exp S$ appears in many fields and thus has different names. It is called *perplexity* in the fields of machine learning and statistical modeling and is a measure of uncertainty in the value of a sample from a discrete probability distribution [3]. The larger the perplexity, the less likely it is that an observer can guess the value that will be drawn from the distribution. It is called *diversity index of order* 1 in the fields of ecology and demography [4]. It is a measure of how many different types (e.g., species) there are in a dataset, i.e., the "effective number of species" in a community.

Shannon entropy is fundamental, but it is not the only useful or natural notion of entropy, even in the context of a single probability distribution on a finite set. Two one-parameter families of entropies include Shannon entropy as a member: the Rényi entropies and the q-logarithmic or Tsallis entropies [4, Figure 4.1].

Both are indexed by a real parameter q, and both have Shannon entropy as the case $q = 1$. Varying q away from 1 can be thought of as deforming Shannon entropy. As in other mathematical contexts where the word 'deformation' is used, the undeformed object (Shannon entropy) has uniquely good properties that are lost after deformation, but the deformed objects nevertheless retain some of the original object's features.

Historically, the first deformation of Shannon entropy was the Rényi entropy family, introduced by Rényi in 1961. The exponential of the Rényi q-entropy is known in ecology as the 'Hill number of order q', denoted by D_q, introduced by Mark Hill in 1973 (acknowledging the prior work of Rényi). In particular, $D_1 = expS$ is the exponential of the Shannon entropy. There is a precise mathematical sense in which the Hill numbers are the only well-behaved measures of diversity [4].

The q-logarithmic entropies have been discovered and rediscovered repeatedly. They seem to have first appeared in a 1967 paper on information and classification by Havrda and Charvát. They were rediscovered in a 1988 paper on statistical physics by Tsallis (without reference to any of the previous rediscoverers). It was after Tsallis that they gained widespread recognition and became most commonly named. The term 'q-logarithmic entropy' is new but has the benefits of being descriptive and of not perpetuating a misattribution [4, Remark 4.1.4].

The q-logarithmic and Rényi entropies have exactly the same content: for each finite value of q, there is a simple formula for one of them in terms of the other, and vice versa. But they have different and complementary algebraic properties. While the Rényi entropies are used to define the diversity indices of order q, the q-logarithmic entropies are used to define the information dimension of order q.

Let be given a finite probability distribution $P = \{p_i : i = 1, \ldots, k\}$, with $0 < p_i < 1$ and $\sum_{i=0}^{k} p_i = 1$ and let $q \in \mathbb{R}$ be a parameter. The *q-logarithmic entropy* of P is defined as

$$S_q = \begin{cases} -\sum_{i=1}^{k} p_i \ln p_i & \text{if} \quad q = 1 \\ \frac{1}{q-1}(1 - \sum_{i=1}^{k} p_i^q) & \text{if} \quad q \neq 1 \end{cases} \tag{C.6}$$

and the *q-Rényi entropy* of P is defined as

$$H_q = \begin{cases} -\sum_{i=1}^{k} p_i \ln p_i & \text{if} \quad q = 1 \\ \frac{1}{1-q} \ln(\sum_{i=1}^{k} p_i^q) & \text{if} \quad q \neq 1 \end{cases} \tag{C.7}$$

Note that both S_q and H_q coincide with Shannon entropy when $q = 1$: $H_1 = S_1 = S$. Moreover, they are both continuous with respect to q, as it can be shown that $\lim_{q \to 1} H_q = \lim_{q \to 1} S_q = S$ (see [4]). Furthermore, the limits $q \to \pm\infty$ always exist and this allows one to define S_q and H_q for $q = \pm\infty$ (see [4]). The relation between S_q and H_q is

$$\exp(H_q) = \exp_q(S_q) \tag{C.8}$$

where the function $\exp_q$ is the *q-deformed exponential*, defined as

$$\exp_q(u) = \begin{cases} \exp(u) & \text{if} \quad q = 1 \\ (1 + (1-q)u)^{\frac{1}{1-q}} & \text{if} \quad q \neq 1 \end{cases} \tag{C.9}$$

In principle, the parameter q can be any real number, but from now on we consider only the case $q \geq 0$.

Let be given a system X equipped with coarse-grained approximation on its state space defined by a sequence of partitions $\mathcal{A}_1 \leq \mathcal{A}_2 \leq \ldots \leq \mathcal{A}_N$. Then we can proceed as before and construct a sequence of probability distributions $P_{\mathcal{A}_N,q}$ and the corresponding sequence of entropies $S_{\mathcal{A}_N,q}$ (defined as in C.2, using formula C.6 instead of C.1). The *information dimension* of order q ($q \geq 0$) of the system X is defined by

$$d_q = \lim_{N \to +\infty} \frac{S_{\mathcal{A}_N,q}}{\ln_q N} \tag{C.10}$$

Her, the function $\ln_q$ is the *q-deformed logarithm*, defined as

$$\ln_q(u) = \begin{cases} \ln(u) & \text{if} \quad q = 1 \\ \dfrac{u^{(1-q)} - 1}{1-q} & \text{if} \quad q \neq 1 \end{cases} \tag{C.11}$$

Again, since the distributions $P_{\mathcal{A}_N,q}$ are univariate, it follows that $0 \leq d_q \leq 1$, for $q \geq 0$.

Since $q \geq 0$, the limit cases are given by $q = 0$ and $q = +\infty$ that are as follows

$$d_0 = 1 \qquad \text{and} \qquad d_{+\infty} = \lim_{q \to +\infty} d_q = 1 \tag{C.12}$$

Now, using relation (C.8) and the fact that the exponential of the Rényi (Shannon) entropy $\exp H_q$ ($\exp S$) is the diversity index or Hill number of order q (1), denoted by D_q, one can show the following deformed version of (C.5): the information dimension d_q is the exponent of the *q-deformed box size scaling* of the average *q-Rényi* information needed to identify an occupied box, that is

$$\exp H_q(\varepsilon) = \exp_q S_q(\varepsilon) \approx [\varepsilon^{-d_q}]_q \qquad \text{for} \quad \varepsilon \to 0^+ \tag{C.13}$$

Again, here $S_q(\varepsilon) = S_{\mathcal{A}_N,q}$ and $H_q(\varepsilon) = H_{\mathcal{A}_N,q}$ is the average Rényi information per box, defined as before. The *q-deformed scaling* is defined by $[\varepsilon^{-d_q}]_q = \exp_q(-d_q \ln_{(2-q)}(\varepsilon))$ (see [4]).

To apply the construction of information dimension to our context, we start by partitioning the genome into N fragments of equal length, where $N = 2, 3, 4, \ldots, N_{\max}$. Denote the set of fragments, i.e., the partition of the genome into N parts, by $\mathcal{A}_N = \{A_1, \ldots, A_N\}$. Hence, if the length of the genome (i.e., the number of sites) is denoted by L, then the length of each fragment of $\mathcal{A}_N$ is approximately $\ell = L/N$. Now we associate a discrete probability distribution $P_N = \{p(A_j) : j = 1, \ldots, N\}$ to each partition $\mathcal{A}_N$ as follows. Let

$$\mu(A_j) = \frac{1}{|A_j|} \sum_{i=1}^{|A_j|} p^i_{\text{comp}}$$

where $|A_j|$ is the number of sites in the fragment A_j and p^i_{comp} is the complementary probability associated to the i-th site of the fragment A_j. Then we define

$$p(A_j) = \frac{\mu(A_j)}{\sum_{j=1}^{N} \mu(A_j)}$$

That is, $p(A_j)$ is the normalized average of the complementary probabilities of each fragment. Now we define the Shannon entropy of the partition $\mathcal{A}_N$ by

$$S_N = -\sum_{j=1}^{N} p(A_j) \ln p(A_j)$$

Now, the 'scaling parameter' of the system is $\varepsilon = 1/N$. Define $S(\varepsilon) = S_N$ and consider the sequence

$$d_\varepsilon = \frac{S(\varepsilon)}{-\ln(\varepsilon)}$$

When ε decreases towards 0 it is expected that the sequence d_ε approximates the information dimension of the DMR. However, as we decrease ε (equivalently, increase N), the fragments A_j become very small, and the associated empirical DMR starts to degrade due to low sampling and the estimate of the information dimension becomes noisier. Then we fit a linear model to the set of computed values of d_ε. If the estimate of the slope is close to zero, i.e., the regression line is essentially horizontal, then it means that the the linear model was capable of averaging out the noise. Now, we can take the estimate of the intercept of the regression line as the estimate of the information dimension d.

References

[1] Petr Danecek et al. “Twelve years of SAMtools and BCFtools”. *Gigascience* 10.2 (2021), giab008.

[2] J Doyne Farmer. “Information dimension and the probabilistic structure of chaos”. *Zeitschrift für Naturforschung A* 37.11 (1982), 1304–1326. DOI: 10.1515/zna-1982-1117.

[3] Fred Jelinek et al. “Perplexity – a measure of the difficulty of speech recognition tasks”. *The Journal of the Acoustical Society of America* 62.S1 (2005), S63–S63. DOI: 10.1121/1.2016299.

[4] Tom Leinster. *Entropy and diversity: the axiomatic approach*. Cambridge university press, 2021. DOI: 10.1017/9781108963558.

[5] Heng Li. “The sequence alignment/map (SAM) format and SAMtools 1000 genome project data processing subgroup”. *Bioinformatics* 25 (2009), 1.

[6] Thomas Minka. *Estimating a Dirichlet distribution*. 2000. URL: https://github.com/tminka/fastfit.

[7] Carl N Morris. “Parametric empirical Bayes inference: theory and applications”. *Journal of the American statistical Association* 78.381 (1983), 47–55. DOI: 10.1080/01621459.1983.10477920.

[8] Sieberth Nascimento-Brito et al. “HIV-1 Tropism Determines Different Mutation Profiles in Proviral DNA”. *PLoS ONE* 10.9 (2015), e0139037. DOI: 10.1371/journal.pone.0139037.

[9] Kai Wang Ng, Guo-Liang Tian, and Man-Lai Tang. *Dirichlet and related distributions: Theory, methods and applications*. John Wiley & Sons, 2011.

[10] Alfréd Rényi. “On the dimension and entropy of probability distributions”. *Acta Math Hungar* 10 (1959), 193–215. DOI: 10.1007/BF02063299.

[11] James T Robinson et al. “igv. js: an embeddable JavaScript implementation of the Integrative Genomics Viewer (IGV)”. *Bioinformatics* 39.1 (2023), btac830.

[12] James T Robinson et al. “Integrative genomics viewer”. *Nature biotechnology* 29.1 (2011), 24–26.

[13] Alexander Robitzsch. *Package ‘sirt’: Supplementary Item Response Theory Models*. 2025. URL: https://CRAN.R-project.org/package=sirt.

[14] Claude E Shannon. “A Mathematical Theory of Communication”. *Bell Sys Tech Journal* 27.3 (1948), 379–423. DOI: 10.1002/j.1538-7305.1948.tb01338.x.

[15] Max Sklar. “Fast MLE computation for the Dirichlet multinomial”. *arXiv:* 1405.0099 (2014). URL: https://github.com/maxsklar/BayesPy.

[16] Nicholas Stoler and Anton Nekrutenko. “Sequencing error profiles of Illumina sequencing instruments”. *NAR genomics and bioinformatics* 3.1 (2021), lqab019.

[17] Jean P Zukurov et al. “Estimation of genetic diversity in viral populations from next generation sequencing data with extremely deep coverage”. *Algorithms for Molecular Biology* 11 (2016), 1–15. DOI: 10.1186/s13015-016-0064-x.

Supplementary Figures

In this appendix, we present three sets of plots. The first (Figure S.1) shows the Histograms of the ln-transformed empirical distributions of mutational rates for the 7 seven experimental conditions. The second (Figure S.2) shows the Q-Q plots of the ln-transformed empirical distributions of mutational rates for the 7 seven experimental conditions. The third (Figure S.3) shows the gaps after excluding sites for the 7 seven experimental conditions.

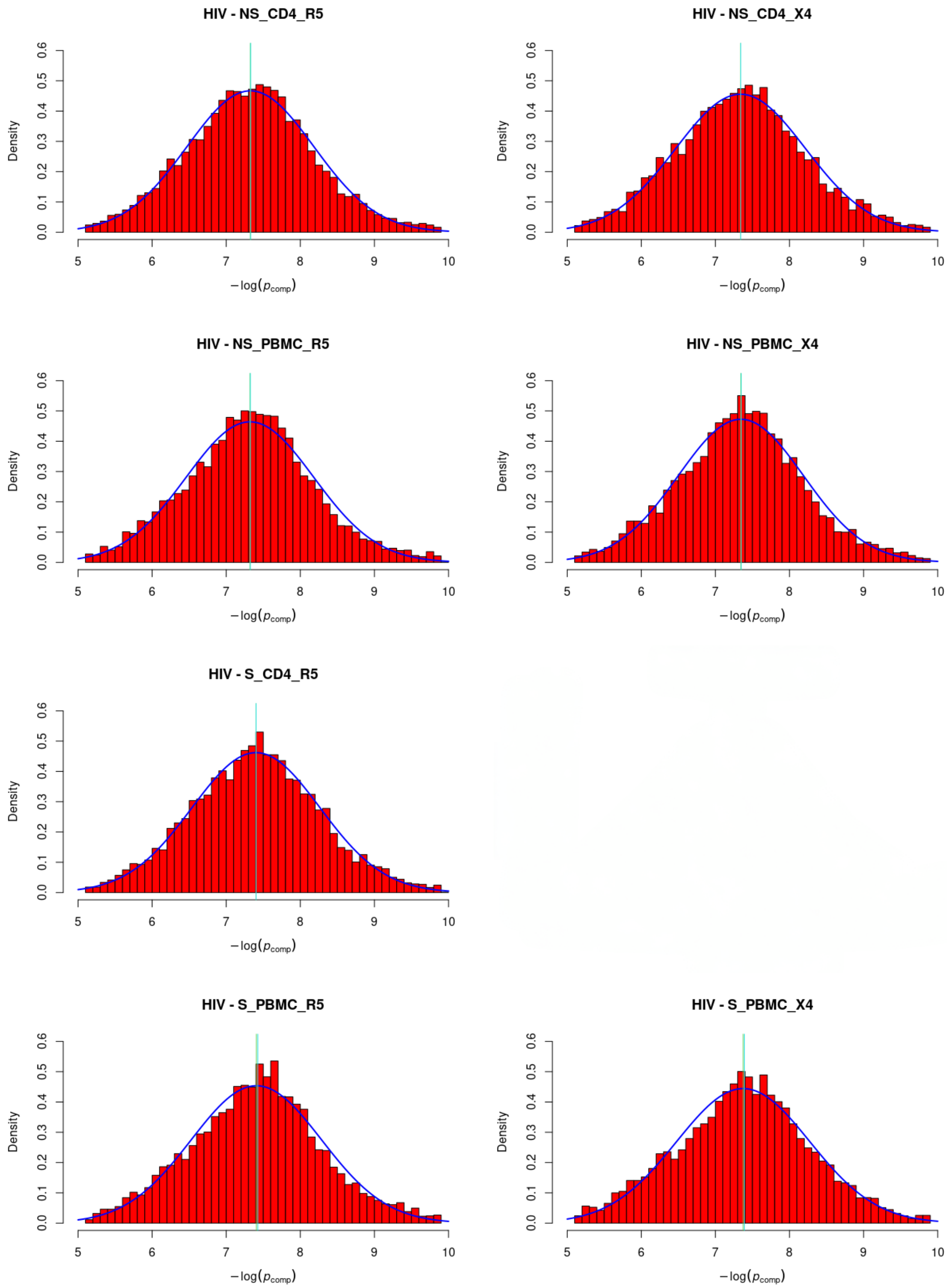

Figure S.1: Histograms of the log-transformed empirical distributions of mutational rates for the 7 seven experimental conditions.

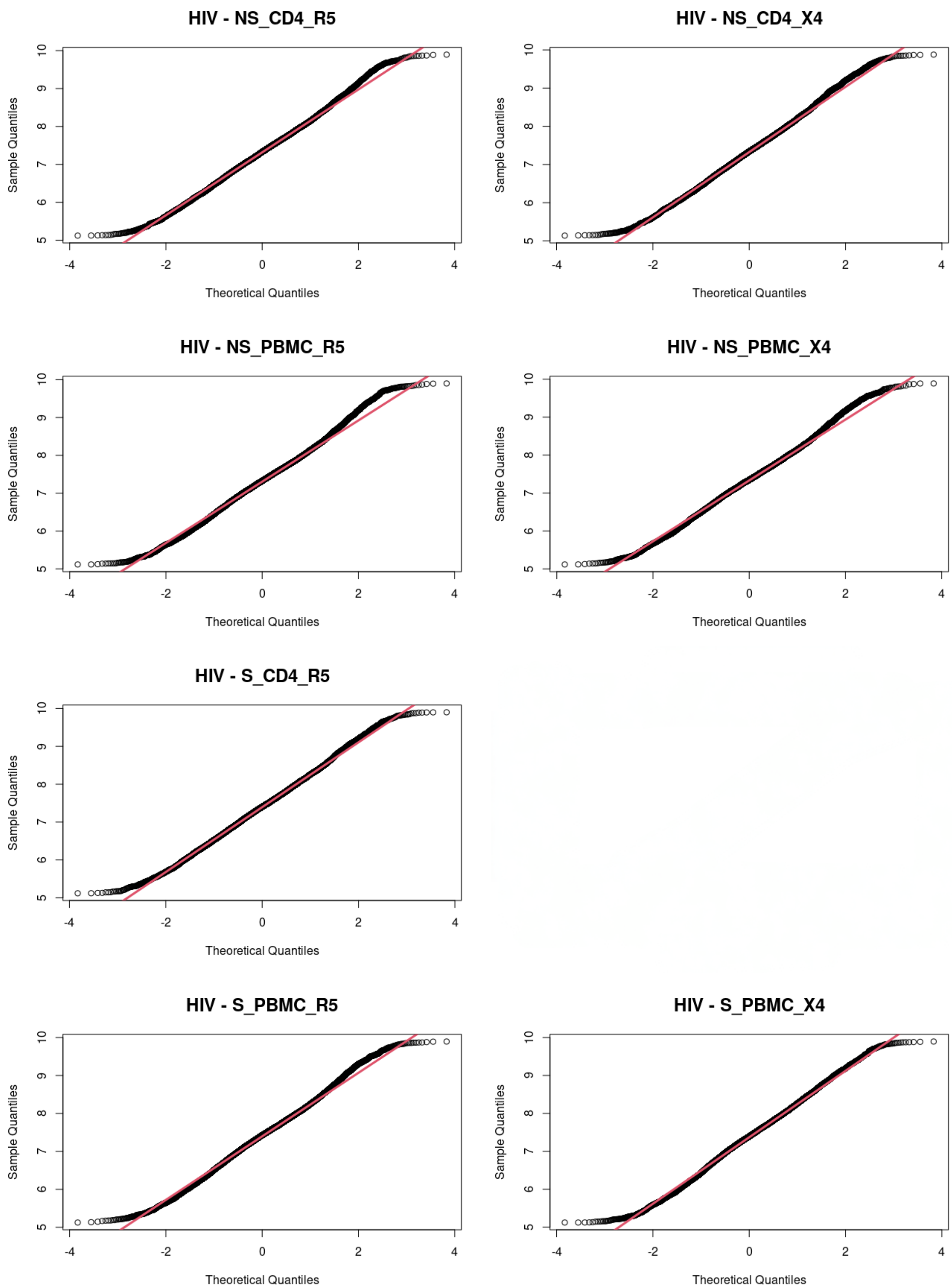

Figure S.2: Q-Q plots of the log-transformed empirical distributions of mutational rates for the 7 seven experimental conditions.

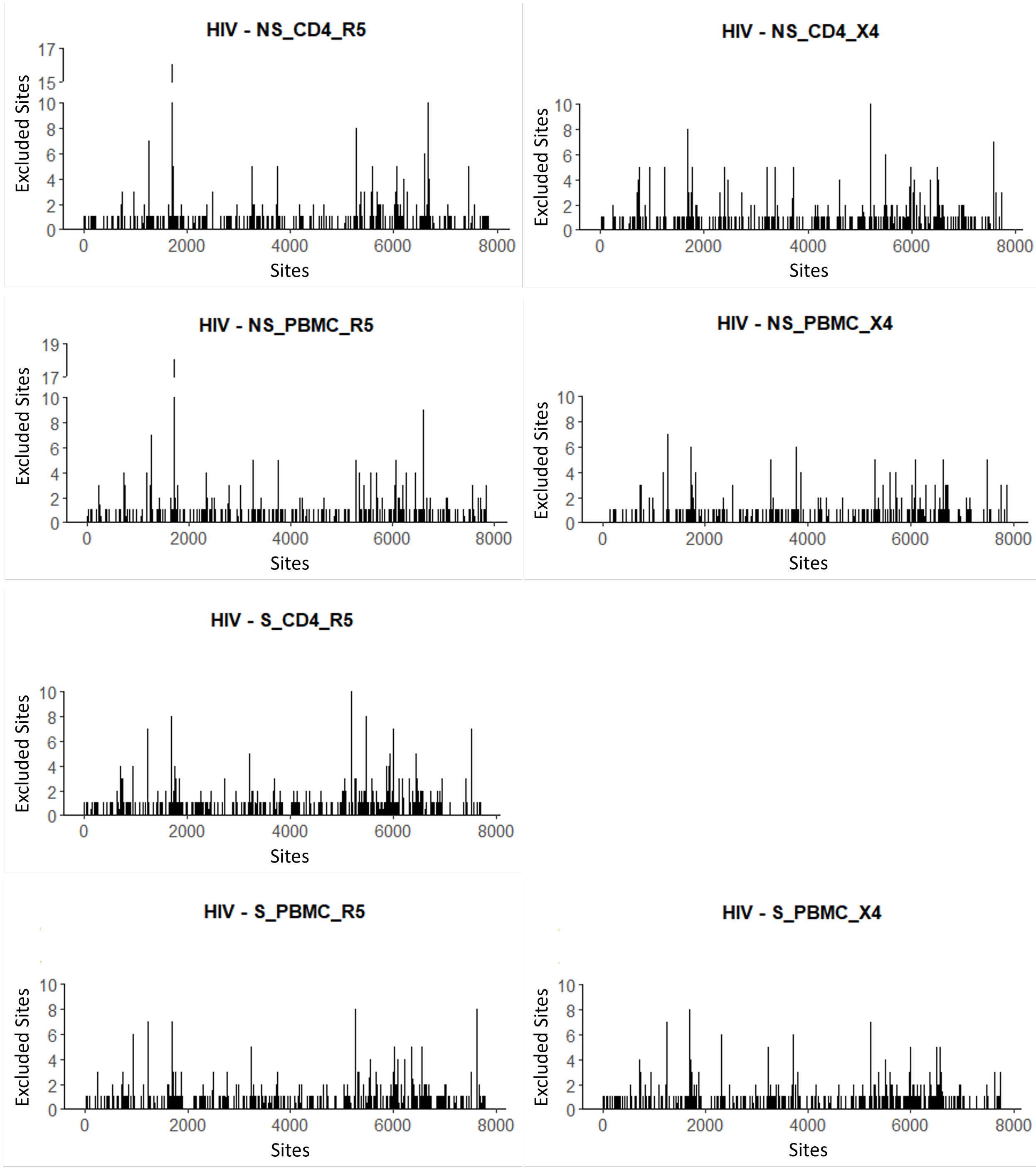

Figure S.3: Gaps positions for the 7 seven experimental conditions. Each bar is the gap length after each non-excluded site. The marks in the x-axis represent the absolute number of non-excluded sites in the specific order and not their location in the genome.
